# Trends in extreme rainfall over the past 55 years suggest springtime subhourly rainfall extremes have intensified in Mahantango Creek, Pennsylvania

**DOI:** 10.1038/s41598-024-79196-3

**Published:** 2024-11-13

**Authors:** Anthony R. Buda, David J. Millar, Casey D. Kennedy, Molly K. Welsh, Adrian R.H. Wiegman

**Affiliations:** 1 Pasture Systems and Watershed Management Research Unit, USDA-ARS, Building 3702, Curtin Road, University Park, PA 16802 USA; 2 Pasture Systems and Watershed Management Research Unit, USDA-ARS, 1 State Bog Road, East Wareham, MA 02538 USA

**Keywords:** Precipitation intensity, Temperature, Long-term trends, Watershed, Climate change, Hydrology

## Abstract

Extreme short-duration rainfall is intensifying with climate warming, and growing evidence suggests that subhourly rainfall extremes are increasing faster than more widely studied durations at hourly and daily timescales. In this case study, we used 55 years (1968–2022) of 5-min precipitation data from Mahantango Creek, a long-term experimental agricultural watershed in east-central Pennsylvania, United States, to examine annual and seasonal changes in subhourly (15-min), hourly, and daily rainfall extremes. Specifically, we evaluated temporal trends in the magnitude and frequency of subhourly, hourly, and daily rainfall extremes. We then estimated apparent scaling rates between rainfall extremes and dew point temperature (Td) and compared these rates to the Clausius-Clapeyron (CC) rate (∼ 7% per °C). We also determined the coincidence of extreme rainfall trends with indicators of atmospheric instability and convective-type precipitation. Overall, we found the most significant changes in rainfall extremes at 15-min durations during the spring, with magnitudes of these subhourly extremes increasing by 0.6 to 0.9% per year, and frequencies rising by 3.4% per year. Apparent scaling rates in the spring showed that 15-min rainfall extremes transitioned from sub-CC scaling to greater than 2CC scaling when Td reached 11° C, implying a possible shift from stratiform rains to more intense convective rains above this Td threshold. Notably, trends in maximum hourly convective available potential energy (CAPE) increased during spring, as did the ratio of 15-min rainfall extremes to their corresponding daily rainfall totals. Findings indicate that convective-type precipitation may be playing an increasing role in the intensification of springtime 15-min rainfall extremes in Mahantango Creek.

**Supplementary Information:**

The online version contains supplementary material available at 10.1038/s41598-024-79196-3.

## Introduction

Extreme precipitation poses considerable challenges to society and the environment. For instance, short-duration (< 1 d) rainfall extremes represent a key ingredient in flash flooding^1^, and areas susceptible to flash flooding are likely to expand as these rainfall extremes intensify with climate change^2–4^. Agriculture is also adversely affected by extreme precipitation, as crop yields are reduced^5,6^and nutrient losses in agricultural runoff are enhanced^7^.

There is strong evidence that rainfall extremes are intensifying worldwide. Many studies document that daily precipitation extremes are rising at global^8–10^and continental^11,12^scales. Studies in the United States (U.S.) also indicate that the magnitude and frequency of daily rainfall extremes are increasing^13–15^. Such observations have supported theoretical expectations that increases in the frequency and magnitude of precipitation extremes are largely attributable to anthropogenic climate warming^11,16,17^.

Climate warming is expected to intensify extreme precipitation because a warmer atmosphere can hold more moisture. This understanding is formalized by the Clausius-Clapeyron (CC) relationship^18,19^, which states that atmospheric moisture increases by about 7% per °C of warming assuming constant relative humidity. Provided that weather circulation patterns remain mostly unchanged, the CC relationship suggests that the amount of moisture available to rainstorms would follow the expected CC rate^20^. Given this theoretical knowledge, many researchers have used observational records to relate rainfall extremes with short-term changes in air temperature or dew point temperature – termed “apparent scaling”^21^. Assuming other atmospheric properties exhibit similar covariance over short and long-term climatic periods, apparent scaling relationships might then provide clues to the possible response of extreme rainfall to climate change – termed “climate scaling”^22^.

Using the apparent scaling approach, a number of global-scale studies suggest that extreme daily rainfall scales with temperature at roughly the CC rate or less^23^. For instance, Westra et al^10^. showed the median intensity of maximum daily precipitation increased with global temperature at rates of roughly 6–8% per °C in line with CC scaling theory. Recent studies have confirmed this finding, showing that daily rainfall extremes generally scale at the CC rate^24,25^. However, other studies of CC scaling at finer spatiotemporal scales have revealed a wide range of apparent scaling rates with temperature, dew point temperature, or a combination thereof^22^.

In particular, short-duration (< 1d) rainfall extremes have deviated appreciably from the CC relation, frequently intensifying at rates less than (sub-CC) or greater than (super-CC) theoretical expectations^22,26^. For example, in Australia, apparent scaling of hourly rainfall extremes followed sub-CC scaling in the northwestern and central regions, but CC and super-CC scaling in the southeastern regions^27^. Elsewhere, regional studies have shown super-CC scaling of hourly extremes in Europe^28^, the U.S.^29,30^ , and Asia^31^, even noting apparent scaling rates of up to three times the CC rate (3CC) in Australia^32^. Studies of subhourly rainfalls have shown apparent scaling rates of nearly double the CC rate (2CC) in the Netherlands^33^and Austria^34^.

A variety of factors may contribute to the shift from sub-CC to super-CC behavior for short-duration extremes. Such factors include changes in atmospheric circulation, moisture availability, and the relative contribution of two major precipitation types – stratiform and convective – to total rainfall^35^. Stratiform rains are generally characterized by cyclonic, large-scale frontal systems that are sustained by forced lifting of airmasses, while convective storms are driven by uplift processes and atmospheric instability that operate over smaller spatial scales^36^. At lower temperatures, stratiform rains of low to medium intensity generally prevail, producing sub-CC to CC scaling. Convective rainfall is typically more responsive to temperature increases than stratiform rainfall^35^. Thus, more intense convective storms regularly dominate at higher temperatures, generating CC to super-CC scaling^35^. In general, the expectation is that short-duration rainfall extremes will intensify faster with warming than daily extremes, as hourly and subhourly rainfall extremes commonly exhibit higher scaling rates with temperature (cf^25^).

Recent studies have confirmed that subhourly rainfall extremes are increasing faster than hourly and daily extremes^37–39^. These studies affirm earlier modeling by Kendon et al^40^. suggesting that changes in 10-min and hourly rainfall extremes would arise before changes in daily extreme precipitation. While a growing number of studies have compared trends in subhourly rainfall extremes to hourly and daily extremes^22,26^, few studies of this nature have been undertaken in the U.S. As such, there is a need for research that evaluates changes in subhourly rainfall extremes relative to more widely reported rainfall durations, with emphases on long-term trends in the magnitude and frequency of extremes^41^, as well as their relationships with temperature. There is also a need to understand the influence of convection on subhourly rainfall extremes, as recent studies in Eurasia have shown that contributions of convective precipitation to total precipitation have risen^42^, especially in the shoulder seasons, which have become more summerlike^36^.

In this case study, we examined annual and seasonal trends in subhourly, hourly, and daily rainfall extremes using 55 years of 5-min precipitation observations from a USDA Agricultural Research Service (ARS) experimental watershed in east-central Pennsylvania, U.S. The 5-min precipitation records analyzed herein are among the longest running records at this temporal resolution in the U.S., making them ideal for studying changes to extreme rainfalls at varying durations, including subhourly durations, which have been relatively understudied in this region. Specifically, we aimed to: (1) characterize the climatology of subhourly, hourly, and daily rainfall extremes, (2) quantify changes in the magnitude and frequency of subhourly rainfall extremes relative to hourly and daily extremes, (3) estimate apparent scaling rates between rainfall extremes and dew point temperature and compare these scaling rates to the Clausius-Clapeyron (CC) rate (∼ 7% per °C), and (4) determine the extent to which significant trends in extreme rainfall are coincident with trends in indicators of atmospheric instability and convective-type precipitation. Findings from this case study have important implications for studies of short-duration rainfall extremes, as well as for agroecosystem management in a changing climate in the central Appalachian region of the eastern U.S.

## Study area

**Fig. 1:**
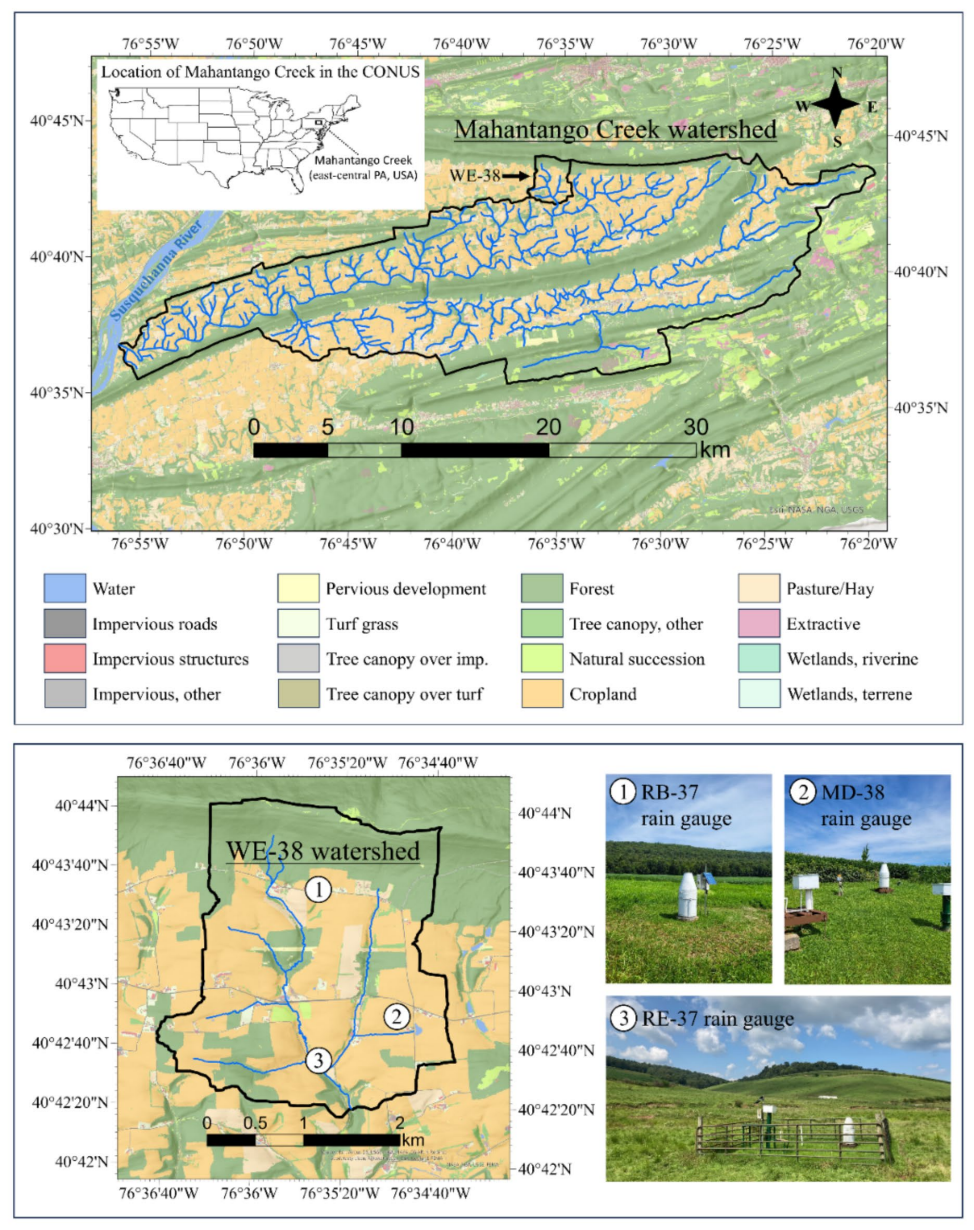
Site map of ARS’s WE-38 experimental watershed in east-central Pennsylvania, U.S. The top panel shows the location of the 420-km^2^ Mahantango Creek watershed within the contiguous U.S. (CONUS), as well as a zoomed-in map depicting the location of the 7.3-km^2 ^WE-38 watershed in the northcentral region of Mahantango Creek. The lower left-hand panel shows the locations of the three permanent rain gauges in WE-38 (1 = RB-37 rain gauge; 2 = MD-38 rain gauge, which is collocated with a climate station; and 3 = RE-37 rain gauge). Also mapped are the Chesapeake Bay Program’s 1-m land use / land cover data^52^, showing mostly cropland and pasture in the valleys, with intact forests confined to the ridges. Maps were created using ArcGIS Pro software (version 3.2.2; ESRI; Redlands, California, U.S.).

The case study was carried out in the 7.3-km^2^ WE-38 experimental watershed, an intensively monitored upland basin in the Ridge and Valley region of east-central Pennsylvania, U.S. (Fig. 1). The WE-38 watershed (outlet: 40°42’16” N, 76°35’16” W) – a headwater basin that is situated in the northcentral region of the 420-km^2 ^Mahantango Creek watershed – has a long record of high-quality hydrometeorological observations^43^that support the study of short-duration rainfall extremes. The watershed has been managed by ARS as a long-term observatory since the late 1960s when ARS’s National Experimental Watershed Network^44,45^was formally authorized by Congress^46^. Since the inception of continuous monitoring in 1968, accumulated precipitation has been recorded every 5 min in the WE-38 watershed^43,47^. Such high-frequency monitoring of precipitation – a hallmark of long-term ARS watershed research (see section S2 in the Supplemental Information) – has produced some of the longest running 5-min precipitation records in the nation. Currently, the WE-38 watershed is one of 23 benchmark experimental watersheds and ranges maintained by ARS across the U.S^48^. The watershed is also part of several national research networks, including the Conservation Effects Assessment Project^49^and the Long-Term Agroecosystem Research (LTAR) network^50,51^.

## Data

### Precipitation measurements

The case study uses continuous 5-min precipitation records from three long-term rain gauging stations in the WE-38 watershed (Fig. 1). Two stations (RB-37 and RE-37) have been operating since 1968, while the third station (MD-38) came online in 1979 (Table 1). From 1968 to 1996/97, all three gauging stations were instrumented with unshielded Fischer and Porter digital punch paper tape weighing rain gauges (a.k.a. Fischer-Porter rain gauges^53–55^). In 1996/97, ARS technicians upgraded the Fischer-Porter rain gauges with load cell datalogging systems, which replaced the digital punch paper systems and weighing mechanisms on the original Fischer-Porter gauges^47^. These upgrades increased the measurement resolution from 2.54 mm under the legacy Fischer-Porter system to 0.254 mm under the newer load cell system. Buda et al^47^. offer more details on rain gauge maintenance and calibration, data processing, and routine quality-control (QC) procedures.

**Table 1:** Information on the three permanent rain gauges in the WE-38 watershed, including time intervals, instrumentation, measurement precision, period of record, and record completeness (also see table S2).

Gauge	Time interval		Fischer-Porter weighing-type rain gauge precision = 2.54 mm			Fischer-Porter load cell rain gauge precision = 0.254 mm			Record Completeness (%)
			Start Date	End Date		Start Date	End Date		
RB-37	5 min		01 Jan 1968	25 Mar 1997		25 Mar 1997	31 Dec 2022		97.6
RE-37	5 min		01 Jan 1968	17 Oct 1996		17 Oct 1996	31 Dec 2022		95.5
MD-38	5 min		01 Jan 1979	10 Oct 1996		10 Oct 1996	31 Dec 2022		99.7

### Record homogenization

Analyses of long-term changes in subdaily precipitation have to account for shifts in instrumentation that affect measurement resolution, as such shifts produce inhomogeneities in the data that cloud the interpretation of trends and event-based statistics^56^. Given the tenfold improvement in measurement resolution that occurred in the WE-38 watershed in 1996/97 (Table 1), it was essential to homogenize the 5-min precipitation time series of each gauge to reflect a common measurement resolution for the entire period of record. To do this, we employed the algorithm of Groisman et al^57^. to convert the finer-resolution measurement period (post 1996/97) to a uniform precision of 2.54 mm for the entire 55-year record (see section S4 and Fig. S4 in the Supplemental Information). This same method has been used to harmonize hourly precipitation data from the National Climatic Data Center’s Hourly Precipitation Data network^25,58,59^, which saw a similar change in precision during its tenure^57^.

### Data aggregation and additional QC procedures

We used RB-37, the longest running and most complete rain gauge record, to generate a nearly serially-complete (i.e., virtually gap free, with missing data < 0.1%) time series of homogenized 5-min precipitation data (see section S3 in the Supplemental Information for more details). We then aggregated the 5-min data to 15-min (subhourly), hourly, and daily intervals. We summarized the data using fixed intervals (e.g., 00:00–00:15, 00:15–00:30 for 15-min data; 00:00–01:00, 01:00–02:00 for hourly data; and 00:00–24:00, 24:00–48:00 for daily data). Data were also separated by meteorological seasons: winter (December-January-February), spring (March-April-May), summer (June-July-August), and fall (September-October-November). Lastly, as an added check on data quality, we subjected the 15-min and hourly time series to additional QC checks using open-source QC algorithms for subhourly (“SubHourlyQC” package^60^) and hourly (“intense-qc” package^61^) rainfall data. These algorithms, implemented in the Python programming environment, automate a series of QC procedures recommended by the World Meteorological Organization (WMO), including constraint, consistency, spike, rapid change, flat line (streak), and domain tests^60,61^.

### Atmospheric reanalysis data

We used two gridded data products from the fifth generation of European ReAnalysis (ERA5) to provide atmospheric data in support of our study. We retrieved daily mean temperature (Ta, °C) and daily mean dewpoint temperature (Td, °C) from the ERA5-Land dataset^62^, as continuous temperature data were not available in WE-38 prior to 1997^63^(see section S6 the Supplemental Information for comparisons between observations and ERA5-Land data). ERA5-Land data are provided at a horizontal resolution of 9 km. In addition, we retrieved hourly data on convective available potential energy (CAPE; J kg^-1^) from the ERA5 global reanalysis^64^, as ERA5 offers some of the most reliable reanalyses of convective parameters^65^. ERA5 data are provided at a horizontal resolution of 31 km. We selected the corresponding grid boxes for ERA5-Land and ERA5 to obtain data for the local region that included the WE-38 watershed. All data were downloaded from the Copernicus Climate Change Service^66^.

## Methods

### Assessing the magnitude of rainfall extremes with block maxima

We used a block maxima approach to assess changes in the magnitude of rainfall extremes^41^. To implement the block maxima approach, we divided the observational periods for 15-min, hourly, and daily rainfall time series into non-overlapping blocks of years and seasons and extracted the largest 15-min (Rx*15min*), hourly (Rx*1h*), or daily (Rx*1d*) rainfall value within each block. The resultant datasets could then be used to evaluate general climatological patterns in rainfall extremes and to estimate trends using nonparametric trend tests like the Mann-Kendall test^41^and extreme value theory^67^.

### Quantifying trends in annual and seasonal rainfall maxima

The block maxima approach enabled us to use two compatible methods for quantifying trends in annual and seasonal rainfall extremes (e.g., Westra et al^10^.). The first method is the nonparametric Mann-Kendall test^68,69^, which has been widely used to evaluate the significance of monotonic trends in hydrometeorological time series^70,71^. A key advantage of the Mann-Kendall test is that it makes no assumptions about the distribution of the data, making it robust to outliers. The second method, which is derived from extreme value theory^67^, fits the nonstationary generalized extreme value (GEV) distribution to annual and seasonal maxima of rainfall extremes, and determines if one (or more) of the three parameters is changing as a function of time or another covariate of interest^72^.

Briefly, we used the R package “modifiedmk”^73^ to execute the Mann-Kendall test and evaluate the significance of monotonic trends in annual and seasonal Rx*15min*, Rx*1h*, and Rx*1d*. The magnitude of the trends was estimated with Sen’s slope estimator^74^. Trends with *p* values less than 0.05 were considered significant.

To conduct nonstationary GEV modeling, we used the R package “extRemes”^75^. The GEV distribution is estimated with three parameters^76^: (1) location (*µ*), which defines the center of the distribution, (2) scale (*σ*), which measures the size of the deviations around the location parameter, and (3) shape (*ξ*), which controls the tail behavior of the distribution. Following the approach of Konstali and Sorteberg^77^, we introduced time as a covariate to the location parameter, while holding the scale and shape parameters constant. We employed the method of maximum likelihood to estimate the GEV parameters. We then used the likelihood ratio test to assess the significance of the time-varying location parameter relative to a stationary model with no covariates. Significant trends in annual and seasonal Rx*15min*, Rx*1h*, and Rx*1d* were inferred when likelihood ratio tests yielded *p* values less than 0.05.

### Quantifying trends in the 99th percentile of rainfall extremes

As noted by Villarini et al^78^., Mann-Kendall tests offer insight into monotonic trends in the center of the data distribution. Using a time-varying location parameter in nonstationary GEV models also focuses on temporal changes in the center of the distribution. To provide a more complete picture of changing rainfall extremes, it is also meaningful to examine changes in other parts of the rainfall distribution using methods like quantile regression^79,80^. Indeed, several recent studies have used quantile regression to assess trends in extreme precipitation^39,77,78,81,82^.

Herein, we used the R package “quantreg”^83^ to assess linear trends in the 99th percentile of annual and seasonal 15-min (P99*15min*), hourly (P99*1h*), and daily (P99*1d*) rainfalls. Unlike the block maxima approach, which utilizes only a small portion of the rainfall data (e.g., the single largest value in a year or season), quantile regression uses the entire rainfall time series. We fitted quantile regression models using a modified version of the Barrodale and Roberts^84^algorithm^85^. Quantile regressions were deemed significant when *p* values were less than 0.05.

### Assessing changes in the frequency of rainfall extremes

There exists a close association between the magnitude and frequency of rainfall extremes, as increases in magnitude are often accompanied by increases in frequency^41^. To test for changes in the frequency of rainfall extremes, we adopted a peaks-over-threshold (POT) approach^14^. To generate annual and seasonal POT time series, we used the 30-year reference period (e.g., Dunn and Morice^86^) from 1971 to 2000 to estimate 99th percentile rainfall thresholds for 15-min, hourly, and daily durations. We included both wet and dry time intervals in the analysis in order to avoid known biases with changes in wet periods^87^. To ensure event independence, we declustered the time series as follows: we retained the maximum 15-min and hourly rainfall amount for each calendar day^32,88^, while a 1-d separation rule was applied to the daily rainfall extremes. Using the declustered time series, we then counted the number of time intervals in the 15-min, hourly, and daily time series that exceeded their respective 99th percentile thresholds.

We used Poisson regression in the R software environment (R Core Team, 2023; version 2.4.3) to evaluate trends in the frequency of rainfall extremes. As noted by Villarini et al^78^. and Mallakpour and Villarini^89^, Poisson regression is a class of generalized linear model (GLM) that is used to model count data^90^, such as the number of extreme rainfall events per year exceeding a high threshold (e.g., Ivancic and Shaw^91^). Poisson regression models were judged significant when *p* values were less than 0.05.

### Scaling of rainfall extremes with dew point temperature

The relationship between extreme rainfall and temperature (a.k.a. “apparent scaling”, or scaling) is essential to understanding how a warming climate influences rainfall extremes^22^. In this study, we used the binning method^28^to analyze apparent scaling relationships between rainfall extremes and Td. We selected Td as a scaling variable over Ta because it is a more reliable indicator of the actual humidity of the air^92^, which has a large effect on scaling^26^. The Glossary of Meteorology^93^defines Td as the temperature a given parcel of air needs to be cooled to at constant pressure to attain a relative humidity of 100%. Thus, a 1 °C rise in Td can be considered roughly equivalent to a ~ 7% rise in atmospheric moisture content^94^. The binning method has been widely applied in the literature (see review by Westra et al^26^.) to analyze the apparent scaling between extreme rainfall and Td (e.g., Ali et al^24^.). Herein, we implemented the binning method using local data on Td from the ERA5-Land model.

To execute the binning method, we compiled separate time series of 15-min, hourly, and daily precipitation using wet intervals only (i.e., intervals with precipitation ≥ 2.54 mm). For each of the time series, we paired each wet interval with its corresponding daily value for Td. Time series of precipitation-Td pairs were constructed for all observations, as well as for seasons. We then arranged the annual and seasonal time series into 20 bins of equal size and sorted the bins from lowest to highest Td. For each bin, we estimated the 99th percentile precipitation amount and the mean Td. We then regressed the logarithm of the 99th percentile precipitation amount onto mean Td. The regression coefficient (*β*) represented the precipitation-Td scaling rate. Using the conversion (*e*^*β*^– 1) × 100%, we expressed the scaling rate as a % change per °C warming^95^.

### Examining the role of convection in extreme rainfall trends

Extreme rainfall events often arise from convective storm systems, especially thunderstorms^96^. These storms are driven by atmospheric instability and moisture availability, which can be formally expressed in terms of CAPE. As such, CAPE provides insight into the potential for convection to influence rainfall extremes^97,98^. Several recent studies have also derived a useful indicator of convection-driven storms that involves estimating a metric called Rx*1h*P, which is defined as the fractional contribution of Rx*1h *to its corresponding daily rainfall total^59,99,100^. A similar calculation can be made for 15-min rainfall extremes, which we denote as Rx*15min*P (see section S7 in the Supplemental Information for more details on the calculations of Rx*15min*P and Rx*1h*P). As Blenkinsop et al^99^. note, values of Rx*1h*P approaching 1 indicate rainfall concentrated in a small part of the day, as would be expected from convective precipitation, while Rx*1h*P values closer to 0 suggest a more even distribution of rainfall throughout the day, as would be expected from stratiform precipitation.

To assess the role of convection in hourly and subhourly rainfall extremes, we assessed whether trends in the annual and seasonal maxima of CAPE were coincident with trends in annual and seasonal Rx*1h*P and Rx*15min*P. We used the Mann-Kendall test and Sen’s slope estimator to evaluate monotonic trends in convective parameters from ERA5. To estimate trends in Rx*1h*P and Rx*15min*P, we employed one-inflated beta regression^101^, which is used to describe rates and proportions that assume values greater than 0 and up to and including 1; beta regression was recently used by Lavers and Villarini^102^to estimate trends in the fractional contribution of atmospheric rivers to total precipitation. One-inflated beta regression models were executed in the R package “gamlss”^103^.

## Results and discussion

### What are the climatological attributes of subhourly, hourly, and daily rainfall extremes?

**Fig. 2:**
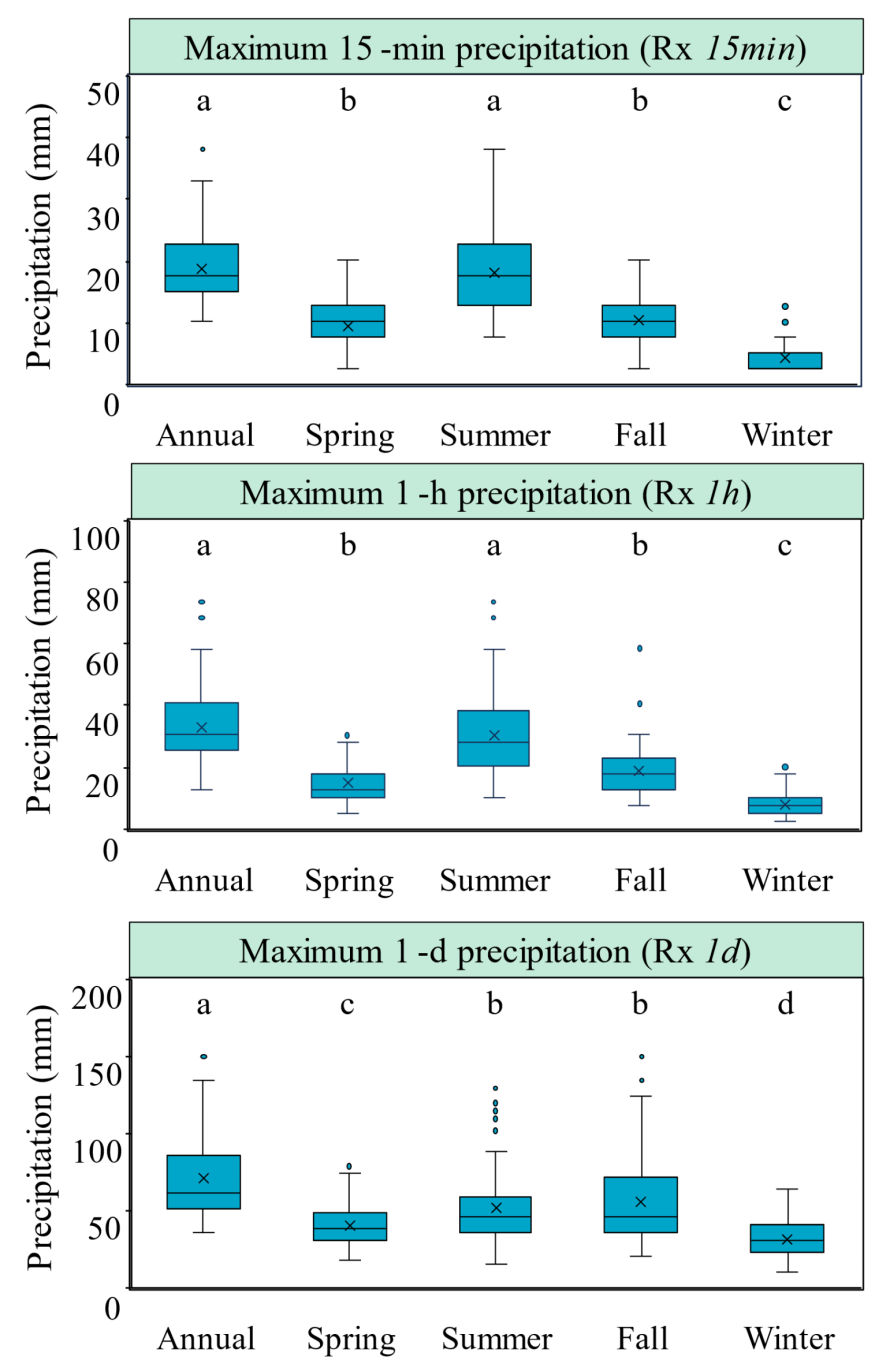
Box plots showing the distribution of annual and seasonal maxima of 15-min (Rx*15min*; top panel), hourly (Rx*1h*; middle panel), and daily (Rx*1d*; lower panel) rainfall in the WE-38 watershed; long-term climatological averages are based on 55 years of data from 1968–2022. For each plot, the box indicates the interquartile range, the whiskers show the extreme values (1.5 times the interquartile range), the asterisks designate potential outliers (values greater or less than three times the interquartile range), the solid line depicts the median, and the “x” indicates the mean. Different letters above each box plot indicate significant differences in the medians based on a Kruskal–Wallis analysis followed by Dunn’s post hoc test (*p* < 0.05). For a comparison of fixed-window vs. moving window results, see Fig. S5 in Supplemental Information.

Prior to conducting trend analyses, we sought to characterize the general climatology of annual and seasonal Rx*15min*, Rx*1h*, and Rx*1d* in the WE-38 watershed. With the exception of summer, seasonal rainfall maxima in WE-38 were generally lower than annual rainfall maxima based on long-term climatological averages (1968–2022) of Rx*15min*, Rx*1h*, and Rx*1d* (Fig. 2). On a seasonal basis, the highest median values of Rx*15min* and Rx*1h* occurred in the summer due to the strong influence of convective precipitation on short-duration rainfall extremes. The highest median values of Rx*1d *occurred in the fall owing to the effects of larger scale, longer duration precipitation events (e.g., stratiform precipitation), including tropical cyclones, which have increased in frequency in the Northeast^104^. The winter months experienced the lowest median values of Rx*15min*, Rx*1h*, and Rx*1d* in the WE-38 watershed.

Here in the U.S., the climatological features of annual Rx*1d* have received considerably more attention than hourly and subhourly rainfall maxima. Therefore, we compared our Rx*1d* results with other studies that have examined Rx*1d *climatologies for the region that included WE-38. In a recent study, Hoerling et al^105^. examined the spatial patterns of Rx*1d* across the U.S. using observations and climate models, with long-term (1948–2018) climatological averages of Rx*1d *ranging from 60 to 70 mm in eastern Pennsylvania. Elsewhere, a global study by Bador et al^106^. also showed that Rx*1d* averaged 60–70 mm in the eastern U.S., albeit with a much shorter period of station-based observations (2001–2013). In the WE-38 watershed, we found that annual Rx*1d* attained a median value of 61.0 mm (IQR = 35.6 mm) over the 55-year study period, which is in line with these national and global scale studies. Unlike Rx*1d*, which is well-studied nationally and globally, long-term data on subdaily rainfall climatologies are currently limited in the U.S. Thus, it was difficult to place our findings for Rx*15min* and Rx*1h *in broader context. That said, this situation is likely to change with the recent advent of global subdaily rainfall datasets^61,107^, which enable the estimation of indices to study these short-duration rainfall extremes^100,108^.

### Is the magnitude of subhourly, hourly, and daily rainfall extremes increasing?

**Fig. 3:**
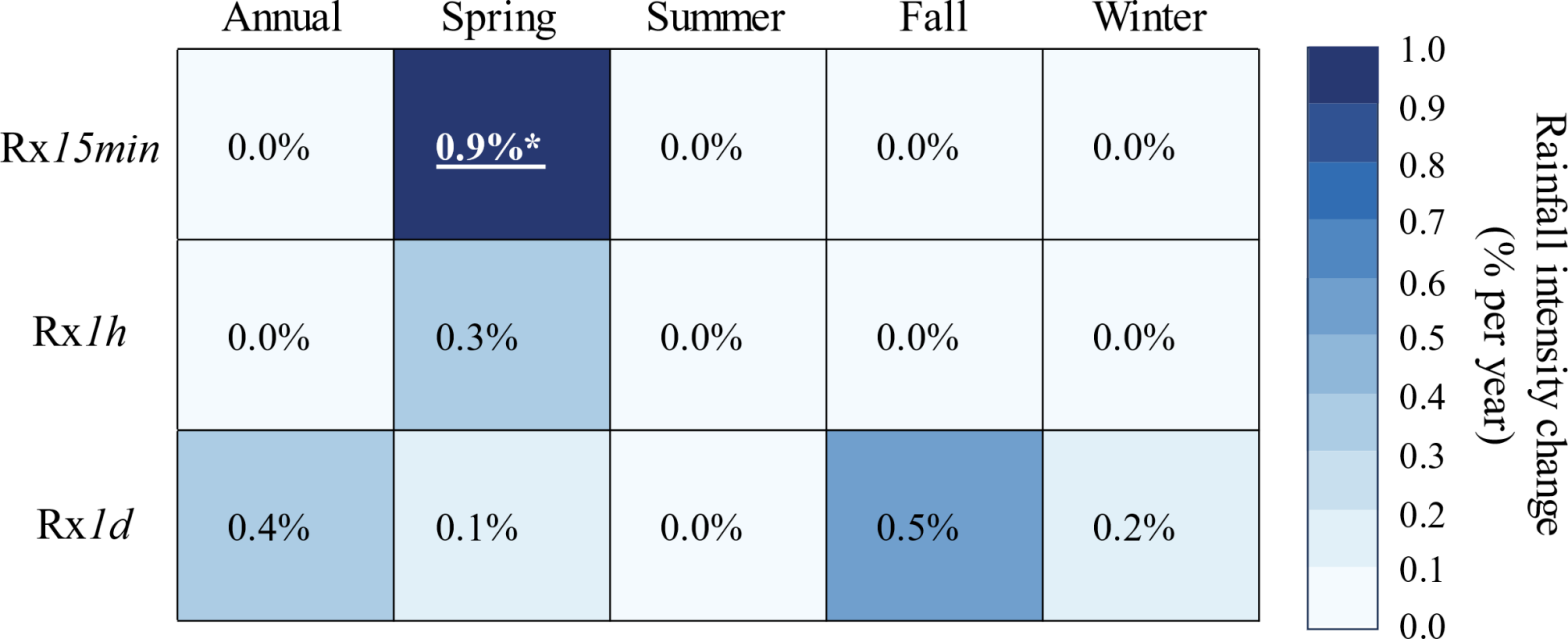
Annual and seasonal trends in the magnitude of Rx*15min*, Rx*1h*, and Rx*1d* based on Sen’s slope. Slopes are normalized over the mean value of each variable and expressed as a percent change per year. Statistically significant trends are determined with the Mann-Kendall test. Significant trends are underlined and bolded, with asterisks indicating the level of significance: * *p* ≤ 0.05; ** *p* ≤ 0.01; *** *p* ≤ 0.001.

**Fig. 4:**
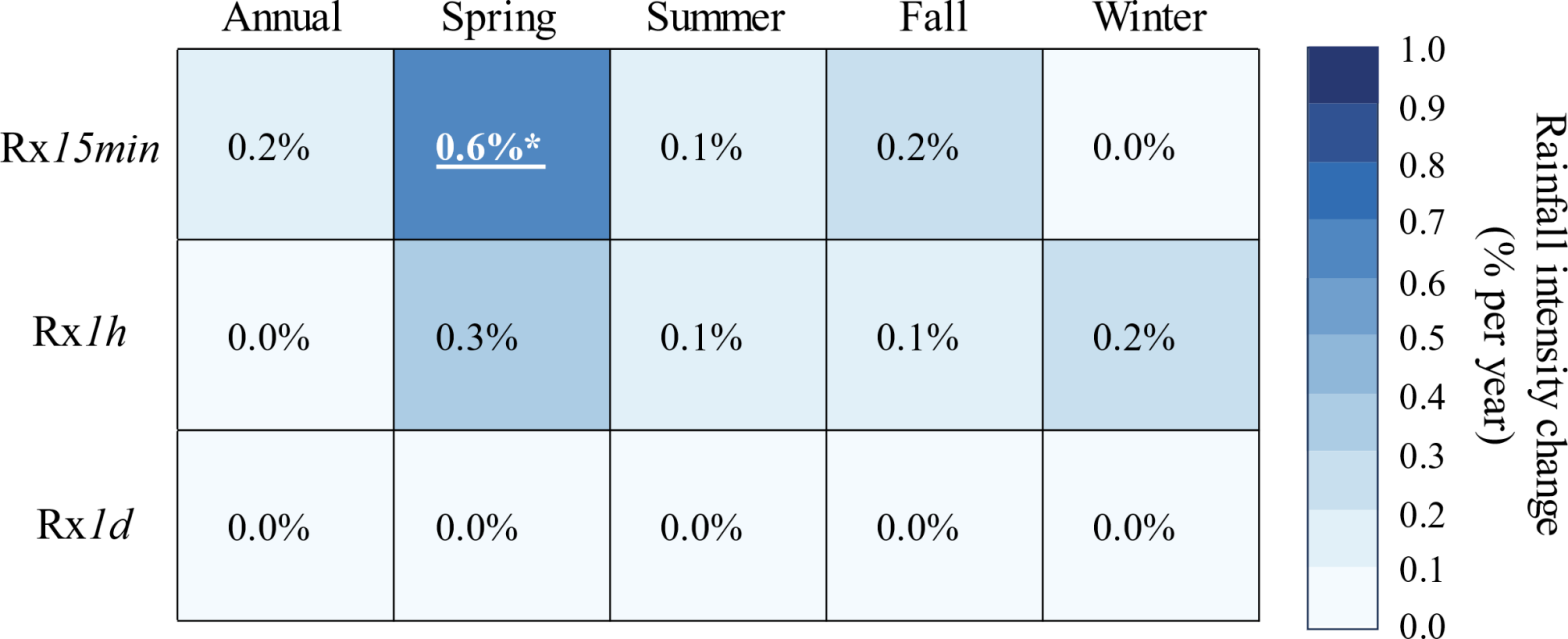
Annual and seasonal trends in the magnitude of Rx*15min*, Rx*1h*, and Rx*1d* using nonstationary GEV models with a time-varying location parameter. Slopes are normalized over the mean value of each variable and expressed as a percent change per year. Statistically significant trends are determined with the likelihood ratio test. Significant trends are underlined and bolded, with asterisks indicating the level of significance: * *p* ≤ 0.05; ** *p* ≤ 0.01; *** *p* ≤ 0.001.

We used the nonparametric Mann-Kendall test and nonstationary GEV models to estimate annual and seasonal trends in Rx*15min*, Rx*1h*, and Rx*1d*. While these tests leveraged fixed-interval data, we also examined moving windows with lengths of 15 min, 1 h, and 24 h, as such aggregation methods can overcome event truncation issues when time steps are fixed^25^. Notably, the results of these tests were mostly consistent for fixed interval and moving window aggregations, therefore, we focused on the fixed window aggregations herein (Figs. 3 and 4) and provided moving window results in supplemental information (Figs. S6 and S7).

Several lines of evidence demonstrate that the most consequential trends in extreme precipitation occurred at subhourly timescales in the spring. For instance, Mann-Kendall tests revealed that spring Rx*15min* increased at a rate of 0.9% per year, which was statistically significant (Fig. 3). Notably, nonstationary GEV models on spring Rx*15min* also showed that the increases in spring Rx*15min* were statistically significant, although the yearly rates of increase (~ 0.6% per year) were slightly lower than those derived from Mann-Kendall tests (Fig. 4).

**Fig. 5:**
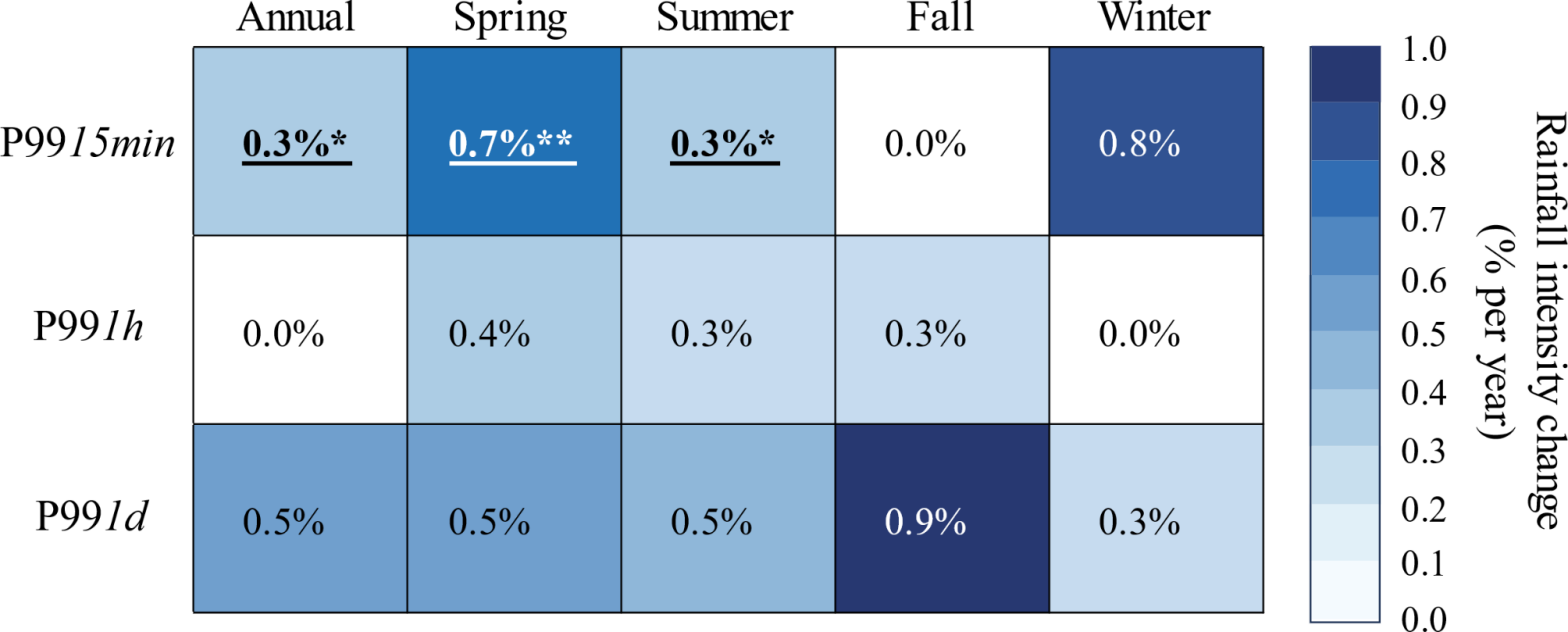
Annual and seasonal trends in the magnitude of P99*15min*, P99*1h*, and P99*1d* based on quantile regression. Regression slopes are normalized over the mean value of each variable and expressed as a percent change per year. Significant trends are underlined and bolded, with asterisks indicating the level of significance: * *p* ≤ 0.05; ** *p* ≤ 0.01; *** *p* ≤ 0.001.

To offer a better sense of changing rainfall extremes in the WE-38 watershed, we also tested for trends in the 99th percentile of the 15-min (P99*15min*), hourly (P99*1h*), and daily (P99*1d*) rainfall time series using quantile regression (Fig. 5). Notably, quantile regression suggested a greater proportion of positive trends relative to the Mann-Kendall tests and nonstationary GEV models (Figs. 3 and 4). Even so, we found noteworthy agreement in terms of which trends were most consequential, as quantile regression models also indicated a statistically significant upward trend in spring 15-min rainfall extremes, with P99*15min* increasing at a rate of 0.7% per year. In addition, quantile regression models also detected statistically significant rising trends in annual and summer P99*15min* (Fig. 5), albeit at slightly lower rates of 0.3% per year. In concordance with the Mann-Kendall tests, quantile regression models found no statistical evidence for changes in hourly or daily rainfall extremes at annual and seasonal timescales.

Our results support the hypothesis that the intensification of subhourly and hourly rainfall extremes might be detected earlier than daily extremes. Indeed, Kendon et al^40^. applied a 1.5-km convection-permitting model to demonstrate that 10-min and hourly rainfall extremes would likely emerge before changes to daily extreme precipitation in the southern U.K. Several observational studies have recently corroborated this assertion. For instance, Ayat et al^37^. employed rain-gauge observations and ground-based radars in the vicinity of Sydney, Australia to show that extreme (99th percentile) and very extreme (99.9th percentile) rainfalls had increased faster at the 10-min resolution than at hourly or daily resolutions. Similarly, Treppiedi et al^39^. determined that 99th percentile 10-min rainfall amounts intensified faster than 99th percentile hourly or daily rainfalls in Sicily, Italy. Most recently, Jayaweera et al^38^. evaluated annual rainfall maxima across a range of storm durations (6 min to 7 d), and showed that the magnitude of short-duration (< 1 h) rainfalls increased faster than longer duration events (> 1 h) for the continent of Australia. Notably, the results of Ayat et al^37^., which were reported for the annual timescale, suggested that extreme and very extreme 10-min rainfalls increased by 0.5 to 2% per year during their 20-year analysis, while Jayaweera et al^38^. reported that the annual intensity of 6- to 18-min rainfalls had increased at rates of roughly 0.4 to 0.6% per year over the course of 55 years. In our 55-year study in the WE-38 watershed, we found equally strong evidence that 15-min rainfall extremes had increased by upwards of 0.9% per year, although the largest increases occurred in the spring season rather than annually as noted by Ayat et al^37^. and Jayaweera et al^38^..

### Are subhourly, hourly, and daily rainfall extremes happening more frequently?

A related objective of our study was to determine if changes in extreme rainfall magnitudes were accompanied by changes in the frequency of rainfall extremes. As such, we adopted a POT modeling approach to ascertain the frequency with which 15-min, hourly, and daily rainfall extremes exceeded their respective 99th percentile thresholds (Table 2).

**Table 2:** Estimates of annual and seasonal 99th percentile rainfall thresholds for 15-min, hourly (1-h) and daily (1-d) durations. Thresholds were estimated for the 30-year reference period from 1971–2000 using declustered 15-min, hourly, and daily rainfall time series.

Duration	99th percentile precipitation threshold, mm (1971–2000)				
	Annual	Spring	Summer	Fall	Winter
15-min	10.2	7.6	15.2	9.4	2.5
1-h	15.2	12.7	23.0	15.2	7.6
1-d	35.6	35.6	40.6	39.9	27.9

**Fig. 6:**
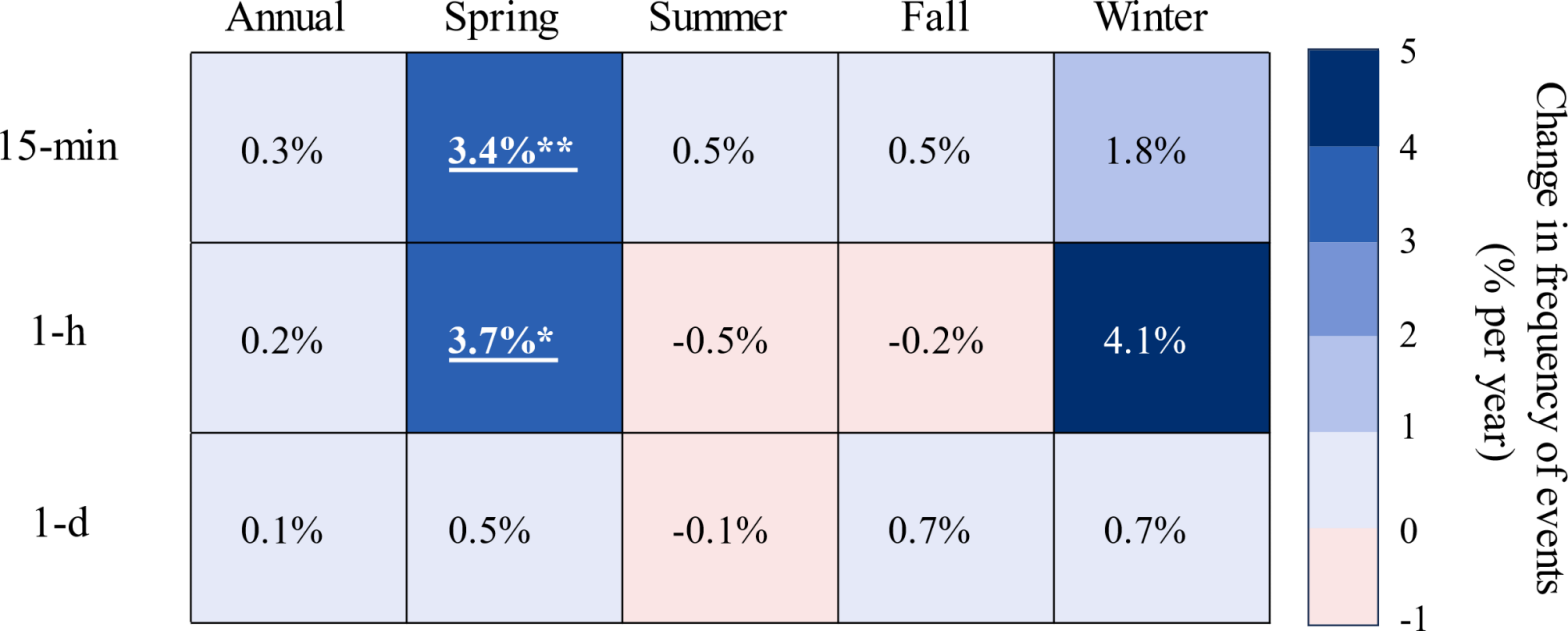
Annual and seasonal trends in the number of events exceeding P9915min, P991h, and P991d intensity thresholds using Poisson regression. Regression slopes are normalized over the mean value of each variable and expressed as a percent change per year. Significant trends are underlined and bolded, with asterisks indicating the level of significance: * *p* ≤ 0.05; ** *p* ≤ 0.01; *** *p* ≤ 0.001.

Using Poisson regression, we found that the spring exhibited a statistically significant upward trend in the frequency of 15-min rainfall extremes (Fig. 6). These extreme events, which increased by 3.4% per year (0.03 events per year), accompanied the increases in extreme 15-min rainfall amounts that were also observed in the spring (Figs. 3, 4 and 5). Notably, the spring experienced an attendant rising trend in the frequency of hourly rainfall extremes, which increased by 3.7% per year (0.03 events per year). Aside from the increasing frequencies of 15-min and hourly rainfall extremes in the spring, we found no additional evidence that the frequency of 15-min, hourly, or daily rainfall extremes was changing at other times of year.

To date, studies that have examined changing frequencies of subhourly and hourly rainfall extremes have been limited relative to those that have assessed daily rainfall extremes. Indeed, there is an extensive literature showing increases in the frequency of daily rainfall extremes at regional^109^, national^14,78,91^, and global^110,111^scales. Recently, however, several studies have indicated that the frequency of hourly rainfall extremes is increasing faster than daily extremes. For instance, Yu et al^112^. used the North American Land Data Assimilation System forcing data to illustrate that increases in the frequency of hourly rainfall extremes outpaced changes in daily extremes, especially in the eastern U.S. More recently, a global study using gridded ERA5 reanalysis data revealed that the frequency of hourly rainfall extremes was increasing nearly two times faster than daily extremes, with some of the largest changes in eastern North America^113^. Our study in the WE-38 experimental watershed lends support to these national and global scale studies of hourly and daily rainfall extremes, showing that increases in the frequency of subhourly and hourly rainfall extremes have emerged well ahead of daily extremes, particularly in the spring. Moreover, the increasing frequency of springtime 15-min rainfall extremes has been accompanied by an attendant increase in the magnitude of these subhourly extremes.

### How does dew point temperature affect subhourly, hourly, and daily rainfall extremes?

**Fig. 7:**
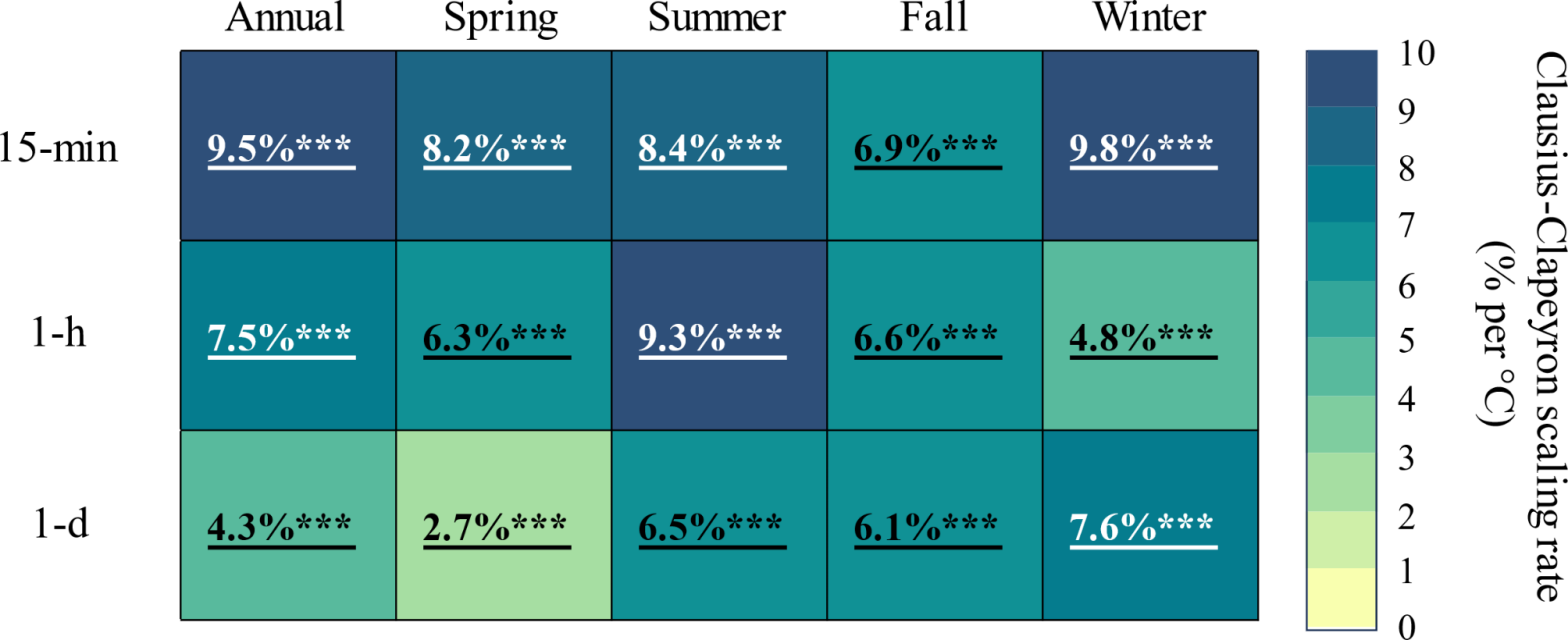
Apparent scaling of annual and seasonal 15-min, hourly (1-h), and daily (1-d) rainfall extremes with dew point temperature (Td, °C). Scaling rates are expressed as a % change per °C of warming. Significant regression relationships between the logarithm of the 99th percentile precipitation amount and mean Td are underlined and bolded. Asterisks indicate the level of significance: * *p* ≤ 0.05; ** *p* ≤ 0.01; *** *p* ≤ 0.001.

**Fig. 8:**
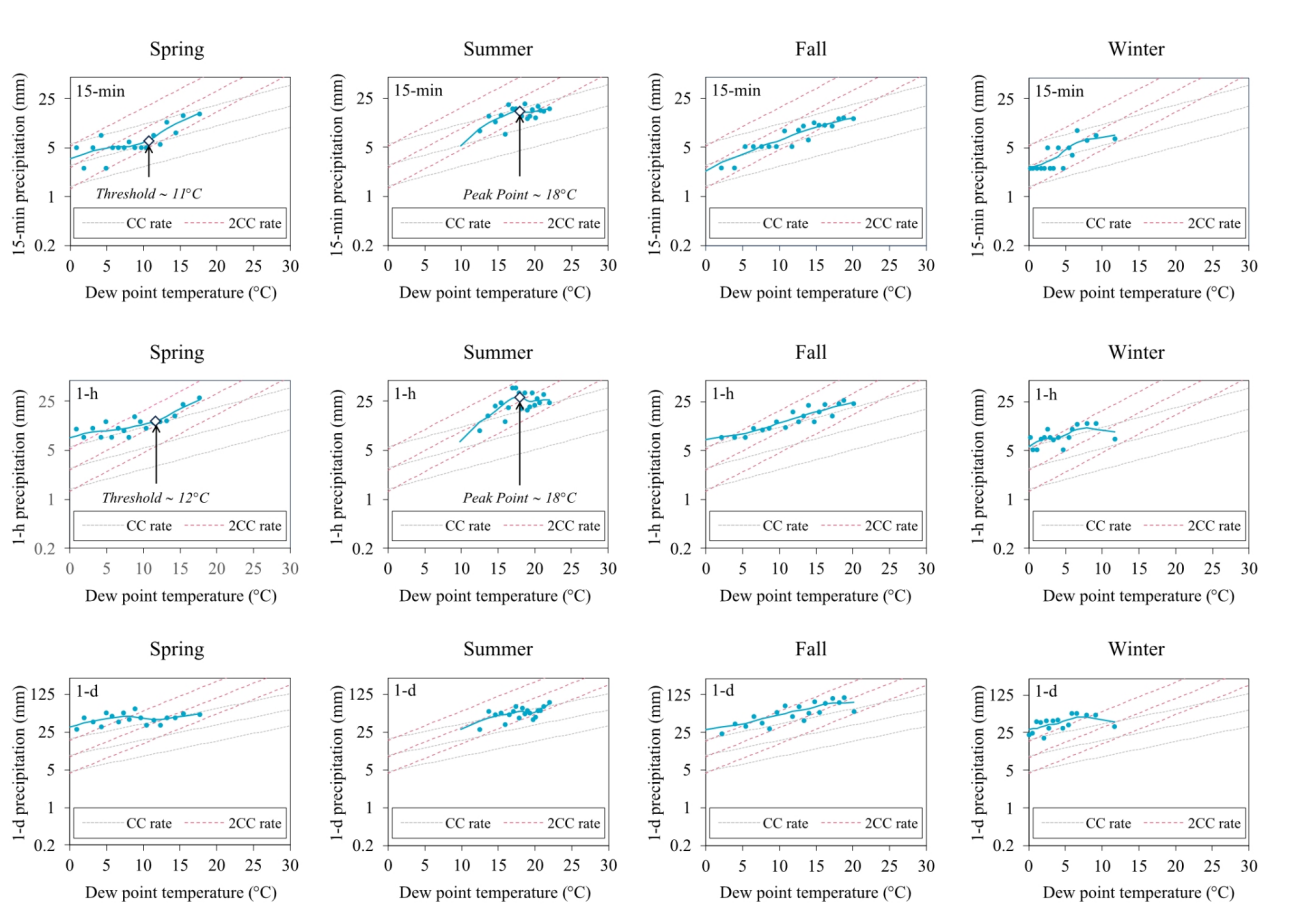
Dependency of 15-min, hourly (1-h), and daily (1-d) rainfall extremes (top to bottom) on dew point temperature (Td, °C) for spring, summer, fall, and winter (left to right). Gray dashed lines indicate the Clausius-Clapeyron (CC) rate of ∼7% per °C, while pink dashed lines demarcate apparent scaling at twice the CC rate (2CC) of ∼14% per °C. Note that the Y-axis for daily scaling plots (bottom row) differs from the Y-axes of 15-min and hourly scaling plots (top and middle rows, respectively).

Increasing temperatures along with changes in atmospheric moisture availability are key factors governing the intensity of extreme rainfall^26^. Such changes are best captured by Td, as it represents the combined effects of temperature and humidity on rainfall intensification processes^22^. Herein, we used the binning method to establish apparent scaling relations between 15-min, hourly, and daily rainfall extremes and daily Td in the WE-38 watershed. While we were generally interested in the overall scaling patterns between 15-min, hourly, and daily rainfall extremes (Fig. 7), we were specifically interested in examining potential shifts in seasonal scaling relations (Fig. 8), as these shifts could offer further insight into the role of precipitation types (e.g., stratiform vs. convective precipitation) on extreme subhourly rainfall trends, especially those that were noted during the spring season.

In terms of overall scaling relations, we found that scaling rates between extreme rainfall and Td generally declined with increasing rainfall duration in the WE-38 watershed (Fig. 7). On an annual basis, 15-min rainfall extremes exhibited super-CC scaling (> ∼7% per °C), with hourly extremes closer to CC rates (∼7% per °C), and daily extremes displaying sub-CC rates (< ∼7% per °C). These findings were generally in line with a study by Schroeer and Kirchengast^34^, who showed that 10-min rainfall extremes in southeastern Austria displayed super-CC scaling, while hourly rates scaled closer to the CC rate, and sub-CC rates prevailed for daily extremes. In the Netherlands, Loriaux et al^33^. also reported that 10-min rainfall extremes attained higher scaling rates with Td than hourly and daily extremes, with nearly 2CC scaling (∼14% per °C) for 10-min rainfalls. We also found that hourly scaling rates generally exceeded daily rates in WE-38, which is consistent with studies that have compared scaling rates among these two durations^114,115^. It is important to note that other studies have reported more similar scaling rates between daily and hourly rainfall extremes using different estimation procedures for apparent scaling (e.g., Bayesian quantile regression, as in Najibi et al^95^.). Even so, super-CC scaling of 15-min rainfall extremes, as was found herein, is in agreement with previous work finding that the intensification of short-duration rainfall extremes generally exceeds thermodynamic expectations of CC scaling (see review by Fowler et al^22^.).

We then evaluated the seasonal scaling patterns in greater detail to assess potential non-linearities in apparent scaling relations that could shed further light on the extreme rainfall trends we observed in WE-38. As Blenkinsop et al^116^. observed, seasonal scaling relationships between extreme hourly rainfalls and temperature are not always linear, and such nonlinearities can offer clues into the types of precipitation that drive rainfall extremes at different times of year. For instance, during summertime in the WE-38 watershed, we found that 15-min and hourly rainfall extremes exceeded 2CC scaling rates (∼ 15 to 19% per °C) up to a Td of ∼ 18 °C, and then leveled off above this threshold (Fig. 8). Such high scaling rates were clearly masked by the overall linear scaling rates indicated in Fig. 7. Convective processes (e.g., thunderstorms) are the prevailing rainfall generating mechanism in the summer, especially for temperatures ranging between 10° and 20 °C^35,94^. As such, the greater than 2CC scaling rates observed for 15-min and hourly rainfall extremes in summer clearly point to convective storms as generators of these extreme rainfalls.

In the spring months, we observed significant increases in the magnitude and frequency of 15-min rainfall extremes (Figs. 4, 5, 6 and 7). More importantly, along with winter, spring was the only other season that exhibited a statistically significant increasing trend in Td (Table S4). In the spring, Td increased 0.02 °C per year, which was generally in line with earlier studies on long-term Td trends in the U.S^117,118^. Given the importance of Td to rainfall intensification processes, we were keenly interested in exploring the extent to which scaling relations in the spring shifted as a function of Td.

Nonlinear apparent scaling relations were clearly evident in spring, as 15-min and hourly rainfall extremes exhibited sub-CC scaling (∼ 5 to 6% per °C) up to Td values of ∼ 11° to 12 °C, and then shifted to greater than 2CC scaling rates (∼ 17 to 18% per °C) above this threshold (Fig. 8). As with the summer, springtime shifts in apparent scaling were concealed by the linear scaling across all values of Td (Fig. 8). A recent study by Blenkinsop et al^116^. in the U.K. also found that hourly rainfall extremes in the spring displayed a threshold-like scaling relation, shifting from sub-CC scaling to super-CC scaling when air temperatures reached 10 °C. According to Haerter and Berg^119^and Berg et al^35^., shifts from sub-CC to super-CC scaling may simply reflect a statistical change in the type of precipitation causing extreme rainfalls from large-scale frontal storms with stratiform precipitation at lower temperatures to smaller scale convective storms at higher temperatures. In an apparent scaling study of hourly rainfall extremes in the U.S., Ivancic and Shaw^29^ suggested that the transition between frontal and convective rains partly explained the super-CC rates they observed in the northeastern U.S. As such, we sought to determine if convection could be playing an increasingly important role in generating springtime rainfall extremes in the WE-38 watershed.

### What is the role of convection in subhourly and hourly rainfall extremes?

**Fig. 9:**
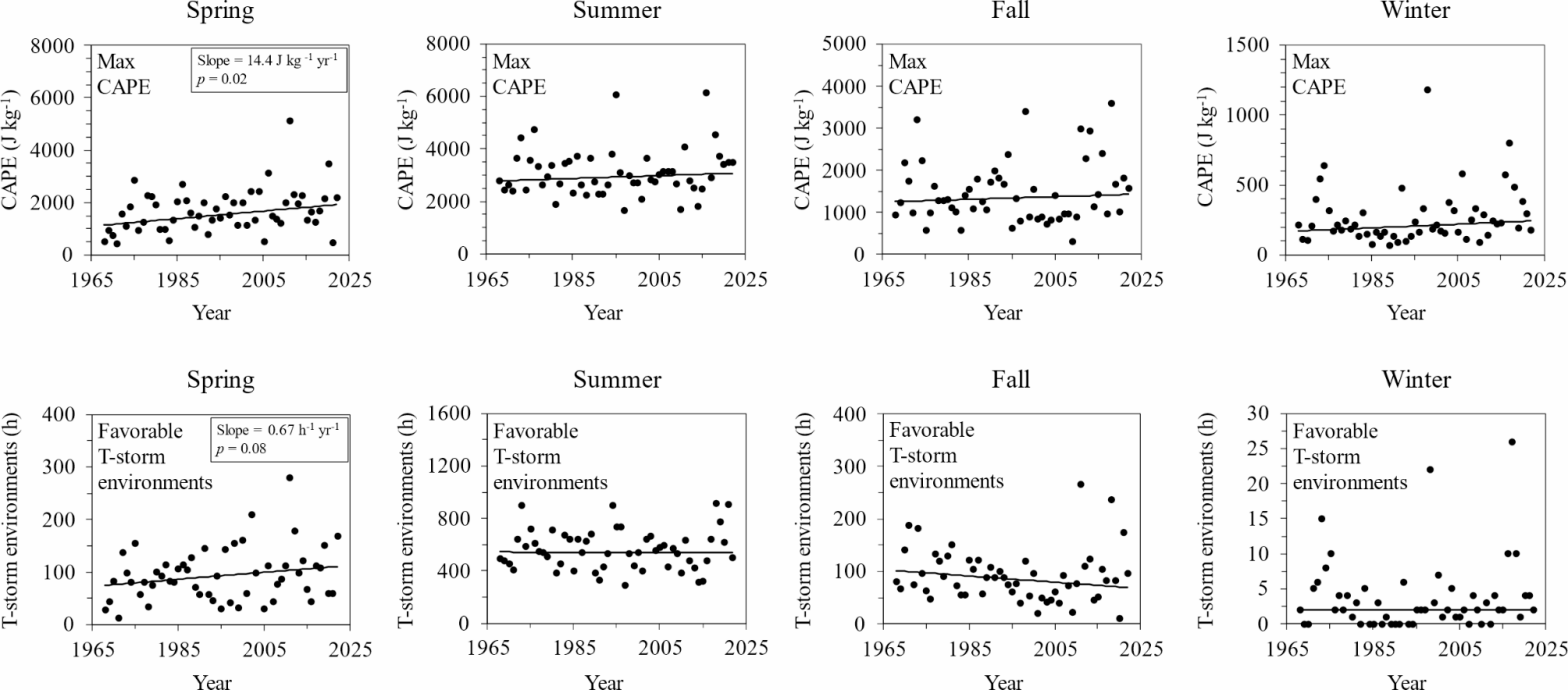
Trends in seasonal maxima of hourly CAPE (top row) and the number of hours per year with favorable thunderstorm environments (bottom row) for spring, summer, fall, and winter (left to right). Best fit regression lines are plotted using Theil-Sen regression. Statistically significant trends are determined with the Mann-Kendall test. Sen’s slope is reported for significant trends, along with the p-value.

We hypothesized that a warming and moistening spring season, as evidenced by the increasing trends in Td (Table S4), might be giving rise to increasingly favorable environments for convection that could be contributing to the intensification of 15-min rainfall extremes in the WE-38 watershed. Indeed, convection plays an important role in the generation of extreme rainfall events^120–122^, and emerging evidence indicates that the length of the thunderstorm season is increasing in the spring and fall due to warming-induced instability^123^. Moreover, recent research also suggests that the frequency and intensity of mesoscale convective systems (organized groupings of thunderstorms) has also risen across central and eastern portions of the U.S^124^. To shed light on this possibility, we used ERA5 hourly CAPE measurements^64^ to assess trends in seasonal maxima of hourly CAPE, as well as the number of hours per year in each season with favorable thunderstorm environments (Fig. 9). Consistent with Taszarek et al^125^., we defined favorable thunderstorm environments as any hour with CAPE exceeding 150 J kg^-1^.

As shown in Fig. 9, spring was the only season with a statistically significant upward trend in CAPE. According to the Mann-Kendall test, maximum hourly CAPE increased by 14 J kg^-1^yr^-1^in the spring, while other seasons showed no noteworthy changes in CAPE. We also found that the number of hours with favorable thunderstorm environments increased by 0.67 h^-1^yr^-1 ^in the spring, although the trend was only significant at the α = 0.10 level. Taken together, these trends suggest that the environment for convective storms has become more favorable in the spring, which may partly explain increases in the magnitude and frequency of 15-min rainfall extremes during this time frame. We note here that increasingly favorable convective environments should not be equated with increases in the frequency or severity of thunderstorms, as CAPE is but one of several ingredients needed for thunderstorm development^125^.

**Fig. 10:**
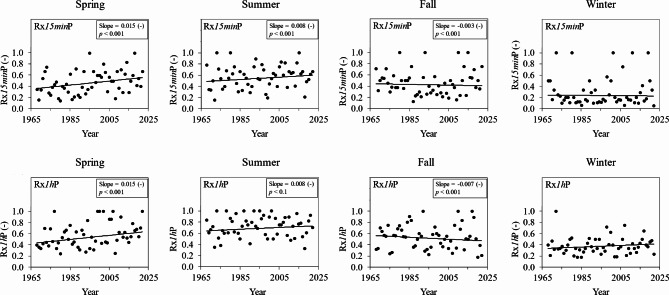
Trends in seasonal Rx*15min*P (top row) and Rx*1h*P (bottom row) for spring, summer, fall, and winter (left to right). Consistent with Barbero et al^59^., we define Rx*15min*P and Rx*1h*P as the fractional contributions of Rx*15min* and Rx*1h* to their corresponding daily rainfall totals. Best fit regression lines are plotted using one-inflated beta regression^101^. Regression slopes are reported for statistically significant trends, along with the p-value. Note that the slopes of beta regression models indicate the change in the log odds of Rx*15min*P or Rx*1h*P being 1 given a unit increase in year.

Considering the potential for increasingly favorable convective environments in the spring, we next explored whether the spring displayed any coincident changes in indicators of convective precipitation (Fig. 10). In accordance with previous studies^59,99,100^, we used Rx*15min*P and Rx*1h*P as indicators of convectively-triggered storms, as these indicators quantify the fractional contributions of Rx*15min* and Rx*1h* to their corresponding daily rainfall totals.

As shown in Fig. 10, spring experienced by far the largest increases in Rx*15min*P and Rx*1h*P, while other seasons showed smaller increases (e.g., Rx*15min*P in summer) and even decreases (e.g., Rx*15min*P and Rx*1h*P in fall). In the spring, the probability of Rx*15min*P being 1 increased from roughly 0.36 in 1968 to 0.55 in 2022, while the probability of Rx*1h*P being 1 rose from nearly 0.44 in 1968 to 0.63 in 2022. Collectively, these trends indicated that 15-min and hourly rainfall extremes in the spring were becoming increasingly concentrated in smaller parts of the day, as would be anticipated if convective precipitation was emerging as the primary driver of these short-duration rainfall extremes. Put another way, 15-min and hourly rainfall extremes in the spring have apparently shifted from contributing less than half of the daily rainfall total in the late 1960s to contributing more than half to nearly two-thirds of the daily rainfall total in the present day. Such evidence suggests an increasingly important role for convection as a trigger for springtime subhourly and hourly rainfall extremes in the WE-38 watershed. These findings are in line with recent studies in Eurasia showing rising contributions of convective precipitation to total precipitation^42^, especially in the shoulder seasons like spring, which have become demonstrably more summerlike with climate warming^36^.

## Limitations and future research needs

Our case study provides important insight into the changing nature of subhourly rainfall extremes in an intensively-monitored agricultural watershed in east-central Pennsylvania, U.S. Indeed, several of the watersheds that are part of ARS’s National Experimental Watershed Network, including the WE-38 watershed, possess some of the longest running 5-min precipitation records in the U.S. These high-quality records enabled the subhourly rainfall trends reported herein using methods that have commonly been applied in the study of hydroclimatic extremes^41^. Even so, we grant that there is ongoing discussion in the literature about the limitations of nonstationary methods of trend testing^126,127^, and that the conclusions we draw from these analyses are strictly germane to the 55-year record we characterized in this study. Moreover, we note that the site-specific nature of the study limited our ability to make definitive statements about the regional significance of these trends^128–130^. While a regionalized analysis was beyond the scope of our case study, future studies should seek to analyze trends across wider geographic areas using larger networks of rain gauges with long-term data on subdaily (or subhourly) rainfall. Regional analyses of subhourly rainfall trends would also offer needed data to test whether subhourly rainfall extremes are systematically intensifying in the spring season, as observed herein, or whether these trends are more locally relevant (e.g., Libertino et al^131^.).

We also recognize that there are alternate approaches to estimating how rainfall extremes scale with Ta and Td^58^. In addition to the binning method used herein^28^, other scaling studies have successfully used quantile regression^132^and the Zhang method for dealing with seasonality^133^. We acknowledge that there is considerable debate in the literature about the most appropriate scaling methods^134–136^, as well as the most informative scaling variables^26^. Moreover, we realize that the apparent scaling estimates obtained in this case study do not necessarily translate to climate scaling estimates. Climate scaling can inform how rainfall extremes might evolve with future climate change^22^, but such estimates are best obtained through broad-scale (regional to global) studies that account for important variations in the atmospheric environment. In our case study, we mainly used the binning approach to obtain useful information about the processes that might have affected apparent scaling relations between subhourly, hourly, and daily rainfall extremes and Td (e.g., Lenderink et al^136^.). Future studies on the apparent scaling of subhourly rainfall extremes with Td would clearly benefit from multimethod (e.g., binning scaling, quantile regression, etc.) regional- to national-scale analyses, such as those used by Ali et al^58^..

Finally, using indicators of convective environments (CAPE) and precipitation triggering mechanisms (Rx*15min*P and Rx*1h*P) represented an indirect way of inferring the role of convective storms in the intensification of subhourly rainfall extremes. Convective storms, or thunderstorms, are rarely monitored directly over long periods of time^137^, and therefore environmental proxies for instability and moisture availability (CAPE) alongside measures of initiation mechanisms like lift are routinely used to assess the ingredients necessary for convection. Other approaches such as radar systems^35^and lightning detection networks^29,138^can be used to obtain information on thunderstorm occurrences, but these data were unavailable for use in this study. Similarly, local observations like storm reports and cloud observations^35,42^ can help to classify rain events as stratiform or convective in nature, but such data were not collected as part of the routine monitoring efforts in the WE-38 watershed. Consequently, leveraging additional sources of data on convective storms (beyond CAPE and proxy variables like Rx*15min*P and Rx*1h*P) would serve to enhance our interpretations regarding the influence of convection on springtime subhourly rainfall extremes.

## Conclusions and implications

In this study, we leveraged 55 years of 5-min precipitation observations in the WE-38 experimental watershed to shed light on annual and seasonal trends in subhourly, hourly, and daily rainfall extremes. We drew the following conclusions from our analyses:

1. *Climatology of extremes*: On average, the highest Rx*15min* and Rx*1h* rainfall amounts occurred in summer due to the preponderance of convective storms in the warm season, while Rx*1d* amounts were highest in the fall, as large scale, stratiform rainfalls typically prevail over convective rainfall types during this time of year. These patterns generally agreed with expectations for daily and subdaily rainfall extremes in the eastern U.S.
2. *Magnitude and frequency*: Over the 55-year study period, the magnitude of 15-min rainfall extremes increased by 0.6 to 0.9% per year in the spring, while the frequency of these springtime extremes also rose by 3.4% per year. The rates at which 15-min rainfalls intensified in the WE-38 watershed were consistent with other studies that have recently examined long-term trends in subhourly rainfall extremes in Europe and Australia. Moreover, the emergence of subhourly rainfall extremes ahead of hourly and daily extremes aligned with recent experimental findings from convection-permitting models.
3. *Effect of Td*: Subhourly, hourly, and daily rainfall extremes scaled strongly with Td. Apparent scaling rates with Td generally declined with increasing rainfall duration, with super-CC scaling for 15-min extremes, CC-scaling for hourly extremes, and sub-CC scaling for daily extremes. Notably, scaling relations in the spring shifted from sub-CC scaling at Td values below 11° C, to greater than 2CC scaling above this Td threshold. This nonlinear pattern indicated a possible statistical shift in the relative importance of stratiform versus convective rains in generating springtime subhourly rainfall extremes.
4. *Role of convection*: The spring season saw upward trends in maximum hourly CAPE and the number of hours per year with CAPE > 150 J kg^−1^, suggesting that the springtime environment had become increasing favorable for convection during the study period. These trends coincided with increasing trends in Rx*15min*P and Rx*1h*P, indicating that 15-min and hourly rainfall extremes were becoming progressively concentrated in smaller parts of the day. Such patterns would be expected if convective precipitation was contributing more to springtime subhourly rainfall extremes than it had in the past.

The implications of our research have relevance to the study of subhourly rainfall extremes, as our investigation adds to the growing number of studies examining extreme subhourly rainfalls in the context of global warming. To date, such studies have been limited due to issues with the quality, accessibility, and coverage (spatial and temporal) of rain gauges with subhourly data^139^. This is especially true in the U.S., where few studies have evaluated trends in subhourly rainfall extremes. While our study offered a local perspective on changes to subhourly rainfall extremes in a long-term experimental watershed, there are opportunities to regionalize and nationalize these findings by leveraging other subhourly rainfall datasets, including those from USDA’s networks of benchmark agricultural watersheds^50^and experimental forests and ranges^140^, as well as the U.S. Climate Reference Network^141^and NOAA’s COOP network^142^. Such studies, along with efforts to collate global subdaily rainfall datasets^143^, will lead to important advances in our knowledge of short-duration rainfall extremes.

From a practical standpoint, findings from this study also have important implications for agricultural interests in the Upper Chesapeake Bay region of the northeastern U.S. A key result of our research was that subhourly rainfall extremes were intensifying faster than hourly and daily extremes in the spring. From an agricultural perspective, spring is an important season for field work, planting, and fertilization, and these activities are often planned around expected precipitation patterns^144^. Increases in the magnitude and frequency of subhourly rainfall extremes, as observed in the WE-38 agricultural watershed, will likely impose challenges to agricultural management. Indeed, extreme rainfalls early in the spring season create a heightened risk for soil erosion, especially if fields are not covered by crops. Extreme rainfalls later in the spring, shortly after crops have been planted, can increase the odds of crop damage^5,6^. Thus, there is an urgent need for additional research on the changing character of subhourly rainfall extremes, as such information will be essential to helping farmers cope with fluctuating weather extremes in a rapidly changing climate.

## Electronic supplementary material

Below is the link to the electronic supplementary material.

Supplementary Material 1

## Data Availability

The datasets generated during and/or analyzed during the current study are available from the corresponding author on reasonable request.
