# Supplementary material for: Trends in extreme rainfall over the past 55 years suggest springtime subhourly rainfall extremes have intensified in Mahantango Creek, Pennsylvania
Source: Sci Rep. 2024 Nov 13;14:27837. doi: 10.1038/s41598-024-79196-3 (PMC11561329; doi:10.1038/s41598-024-79196-3)
Supplement: Supplementary file 1 — Supplementary Material 1 [file 41598_2024_79196_MOESM1_ESM.docx]

Supplementary Material

*Scientific Reports*

**Trends in extreme rainfall over the past 55 years suggest springtime subhourly rainfall extremes have intensified in Mahantango Creek, Pennsylvania**

Anthony R. Buda,^a^ * David J. Millar,^b^ Casey D. Kennedy, ^b^

Molly K. Welsh,^b^ and Adrian R.H. Wiegman,^b^

^a^ USDA-ARS, Pasture Systems and Watershed Management Research Unit, Curtin Road, University Park, PA 16802

^b^ USDA-ARS, Pasture Systems and Watershed Management Research Unit, East Wareham, MA 02538

* *Corresponding author*: Anthony R. Buda, [Anthony.Buda@usda.gov](mailto:Anthony.Buda@usda.gov)

S1. Introduction

This supplementary material includes supporting figures and tables, as well as additional analysis and interpretation related to the study of subhourly, hourly, and daily rainfall extremes in the Mahantango Creek watershed. Specific topics addressed include: (1) uniqueness of the 5-min precipitation records in the Mahantango Creek experimental watershed, (2) methods for infilling missing data and generating a serially complete record, (3) effects of homogeneity adjustments on 5-min precipitation data, (4) comparisons of fixed interval and moving window aggregations on trends in Rx*15min*, Rx*1h*, and Rx*1d*, (5) air temperature and dew point temperature data and trends, and (6) impacts of ties on estimations of Rx*15min*P and Rx*1h*P.

S2. Uniqueness of the 5-min precipitation records in Mahantango Creek

The availability of continuous, high-quality data on precipitation and watershed runoff is an outgrowth of USDA’s enduring commitment to long-term hydrologic research. In the late 1960s, Congress authorized permanent funding for USDA’s Agricultural Research Service (ARS) to launch six regional hydrology research centers in representative hydroclimatic regions of the U.S.^1^. These centers supplemented existing research watersheds in Texas and Ohio, which had been operating since the Dust-Bowl era of the mid-1930s. Today, the ARS experimental watershed network consists of ten primary locations across the U.S. that routinely monitor hydroclimate, water quality, and land management^2^.

A hallmark of many of ARS’s long-term experimental watersheds, including Mahantango Creek, is the systematic measurement of subhourly precipitation. Many of the longest-running locations in the ARS experimental watershed network possesses historical records of subhourly precipitation dating back to the mid-1930s, with precipitation measurements reported as frequently as once per min but no less frequent than once every 15 min (Table S1).

*Table S1. Subhourly precipitation records maintained by ARS’s long-term experimental watershed network. Details on the precipitation records from ARS’s Mahantango Creek experimental watershed – which formed the basis for the current study – are highlighted in blue.*

| **ARS Experimental Watersheds** | | | |  | **Subhourly precipitation observations** | | |
| --- | --- | --- | --- | --- | --- | --- | --- |
| **Watershed** | **Research unit** | **Location** |  | | **Period of record** | **Finest time step** | **Reference** |
| Riesel | Grassland Soil and  Water Research Laboratory | Riesel,  TX |  | | 1938 – present | 5 min | D. Harmel et al.^3^ |
| North Appalachian | North Appalachian  Experimental  Watershed | Coshocton, OH |  | | 1938 – 2012 | 1 min | Bonta^4^ |
| Walnut Gulch | Southwest Watershed  Research Center | Tucson,  AZ |  | | 1953 – present | 1 min | Goodrich et al.^5^ |
| Little Washita River | Grazinglands Research Laboratory | El Reno, OK |  | | 1962 – present | 5 min | Starks et al.^6^ |
| Reynolds Creek | Northwest Watershed  Research Center | Boise,  ID |  | | 1962 – present | 15 min | Hanson^7^ |
| Little River | Southeast Watershed  Hydrology Research Laboratory | Tifton,  GA |  | | 1967 – present | 5 min | Bosch et al.^8^ |
| Mahantango Creek | Pasture Systems and  Watershed Mgmt.  Research Unit | Univ. Park, PA |  | | 1968 – present | 5 min | Buda et al.^9^ |
| Goodwater Creek | Cropping Systems and Water Quality Research Unit | Columbia, MO |  | | 1969 - present | 2 min | Sadler et al.^10^ |

When the Mahantango Creek watershed was initiated in 1966, early research focused on assessing the spatiotemporal variation of precipitation throughout the 420-km^2^ watershed using an intensive network of 43 rain gauges that measured precipitation at 5 min intervals^11^. Changes in funding and research priorities led to reductions in the size of the network^9^, and by 1976, only two permanent rain gauges remained: RB-37 and RE-37 (Fig. 1). These gauges were located in the 7.3-km^2^ WE-38 watershed (Fig. 1), which became the focus of the unit’s hydrologic research program from 1976 onward^12^.

Continuous 5-min precipitation records in the WE-38 watershed originate from the two rain gauges that were part of the initial network (RB-37 and RE-37) as well as a third gauge (MD-38) that came online in 1979^9^. Remarkably, the two longest-running rain gauges (RB-37 and RE-37) have precipitation records that are at least 95% complete, while the more recent gauge (MD-38) possesses a record that is nearly 100% complete (Table S2).

*Table S2. Information on record completeness for the three permanent rain gauges in the WE-38 watershed. Summaries are provided for eleven five-year periods from 1968–2022, as well as for the period of record.*

| **Years** | **RB-37** | | |  | **RE-37** | | |  | **MD-38** | | |
| --- | --- | --- | --- | --- | --- | --- | --- | --- | --- | --- | --- |
|  | **Days Missing** | **Total Days** | **Percent Missing** |  | **Days Missing** | **Total Days** | **Percent Missing** |  | **Days Missing** | **Total Days** | **Percent Missing** |
| 1968–1972 | 28.5 | 1827 | 1.6% |  | 37.2 | 1827 | 2.0% |  | --- | --- | --- |
| 1973–1977 | 10.4 | 1826 | 0.6% |  | 16.6 | 1826 | 0.9% |  | --- | --- | --- |
| 1978–1982 | 151.7 | 1826 | 8.3% |  | 80.7 | 1826 | 4.4% |  | 2.3 | 1461 | 0.2% |
| 1983–1987 | 245.9 | 1826 | 13.5% |  | 652.6 | 1826 | 35.7% |  | 7.6 | 1826 | 0.4% |
| 1988–1992 | 0 | 1827 | 0.0% |  | 28.8 | 1827 | 1.6% |  | 0 | 1827 | 0.0% |
| 1993–1997 | 32.3 | 1826 | 1.8% |  | 9.8 | 1826 | 0.5% |  | 0 | 1826 | 0.0% |
| 1998–2002 | 0 | 1826 | 0.0% |  | 59.1 | 1826 | 3.2% |  | 26.1 | 1826 | 1.4% |
| 2003–2007 | 5.3 | 1826 | 0.3% |  | 16.3 | 1826 | 0.9% |  | 2.7 | 1826 | 0.1% |
| 2008–2012 | 0 | 1827 | 0.0% |  | 0 | 1827 | 0.0% |  | 0 | 1827 | 0.0% |
| 2013–2017 | 0 | 1826 | 0.0% |  | 0 | 1826 | 0.0% |  | 0 | 1826 | 0.0% |
| 2018–2022 | 0 | 1826 | 0.0% |  | 0 | 1826 | 0.0% |  | 9.9 | 1826 | 0.5% |
| 1968–2022 | 474.1 | 20089 | 2.4% |  | 901.1 | 20089 | 4.5% |  | 48.6 | 16071 | 0.3% |

To demonstrate the uniqueness of the 5-min precipitation data from the WE-38 watershed, we briefly review the record lengths and completeness statistics of subhourly precipitation datasets that are currently available from active rain gauging networks in the northeastern U.S. Two such networks, which are operated by NOAA’s National Centers for Environmental Information (NCEI), include: (1) COOP-Hourly Precipitation Data (HPD)^13,14^, which is derived from raw 15-min measurements, and (2) U.S. Climate Reference Network (USCRN^15^), which measures climate variables every 5 min. While subhourly precipitation records are also provided by NCEI’s Automated Surface Observing Systems (ASOS), we did not consider these data because ASOS is mostly focused on meeting aviation forecast needs^16^ rather than supporting long-term climate monitoring^17^. Indeed, there are notable discontinuities in ASOS data^17^, including precipitation measurements^18^, which create challenges for using these data in long-term studies of climate variability and trends. The presence of such discontinuities could partly explain why ASOS records were not included in the Global Sub-Daily Rainfall Dataset recently compiled by Lewis et al.^19^ and summarized by Pritchard et al.^20^.


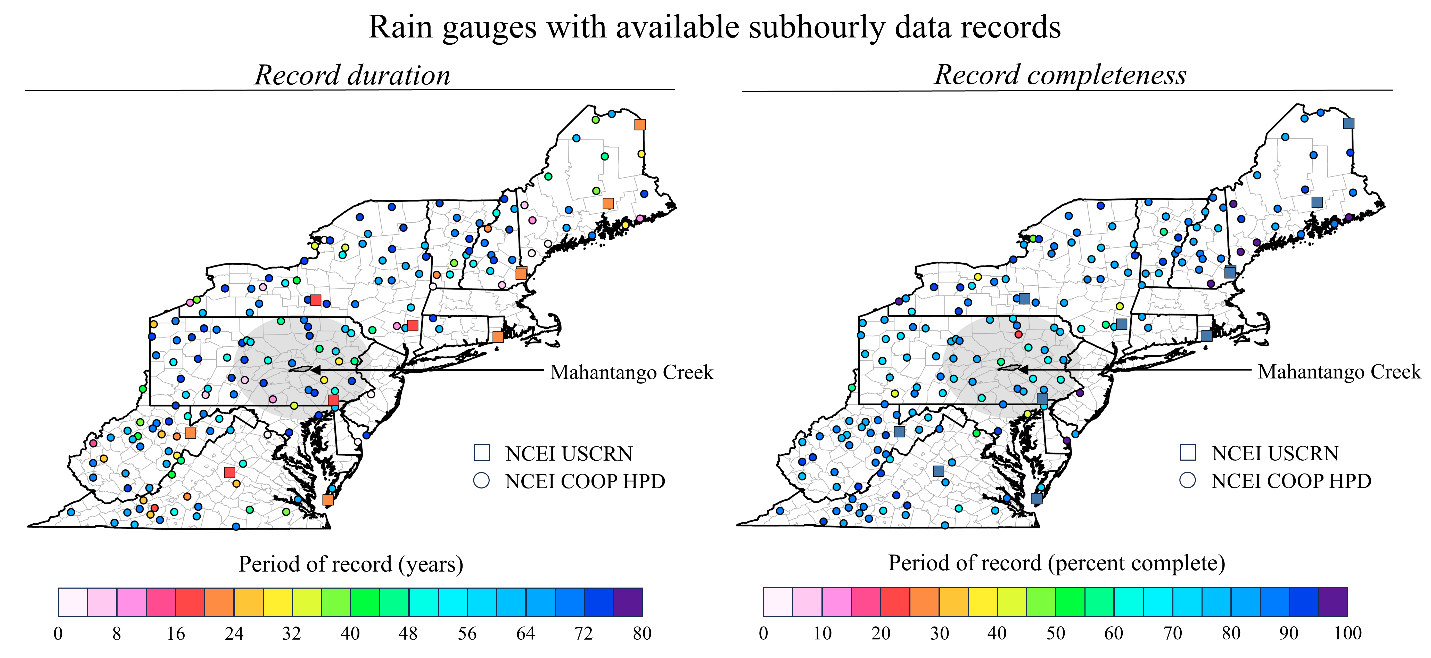


*Fig. S1. Maps showing record duration (left panel) and record completeness (right panel) for active rain gauging stations that contribute to NCEI’s COOP Hourly Precipitation Data (HPD) and Climate Reference Network datasets in the northeastern U.S. The grey circle is used to show stations that lie within a 150-km radius of ARS’s long-term rain gauges in the Mahantango Creek watershed. The 150-km radius is a rough approximation of the correlation distance for daily rainfall extremes^21,22^.*


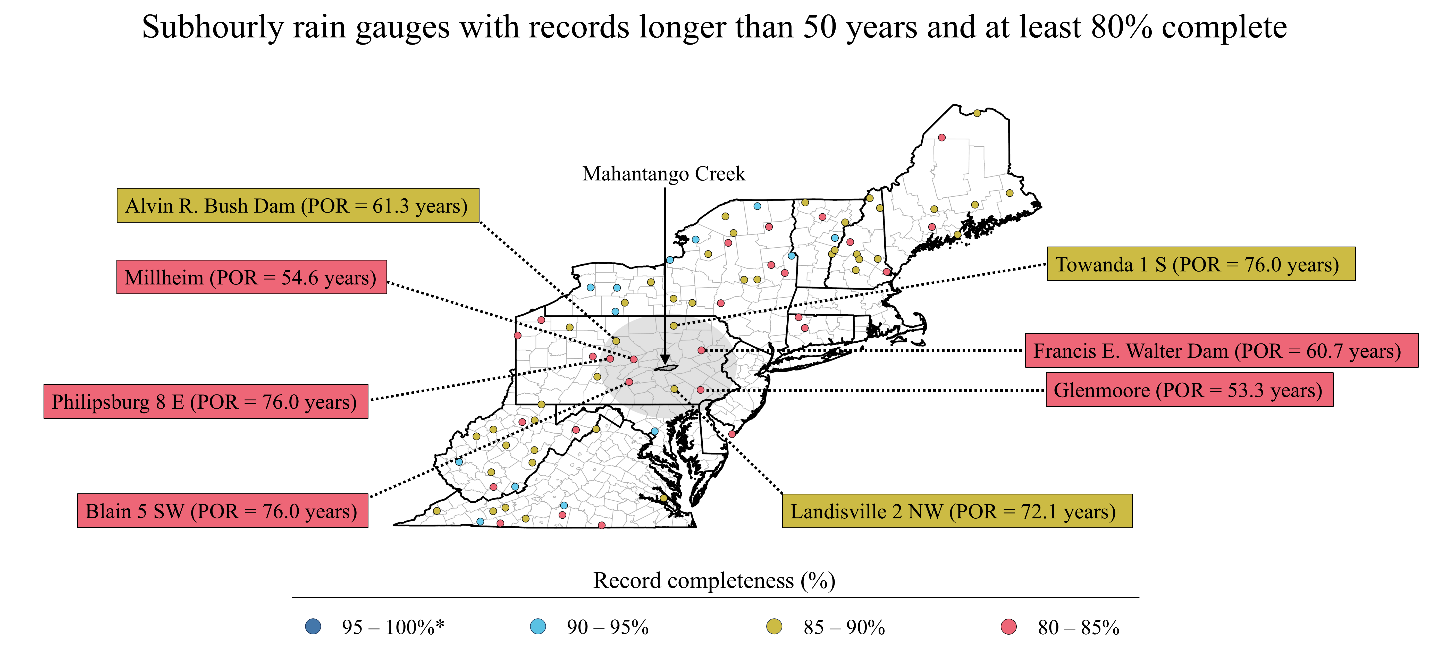


*Fig. S2. Map showing the locations of subhourly rain gauges in the northeastern U.S. with at least 50 years of precipitation data and a record completeness of at least 80%. A 50-year record is chosen as the cutoff for record length, as 50 years of observations is deemed necessary for detecting trends that may be related to climate change^23^. A record completeness of 80% is chosen because this is typically the minimum level of completeness that researchers use for including rain gauges in assessments of long-term trends. The gray circle is the same as in Fig. S1; POR = period of record.*

As shown in Fig. S1, the northeastern U.S. has 12 USCRN stations and 184 HPD stations that provide subhourly precipitation data. Notably, all USCRN stations measure precipitation amounts every 5 min, which is commensurate with the frequency of precipitation measurement in the WE-38 watershed. In addition, USCRN records are nearly 100% complete. Unfortunately, the record lengths of USCRN stations range from 17–22 years, which remain too short for long-term trend studies^23^. In contrast, the record lengths of HPD stations tend to be much longer, with 66% of the stations having records longer than 50 years. However, record completeness for these HPD stations ranges widely, from 23–94%.

There is a lack of consensus on the level of record completeness deemed necessary for including rain gauges in long-term trend studies of precipitation extremes. While some studies are willing to tolerate as much as 20% missing data^24^, others require no more than 5% missing data^25^. In Figure S2, we show the HPD rain gauges in the northeastern U.S. with at least 50 years of data and no more than 20% missing data. Across the entire northeastern U.S., there are 76 HPD stations that meet these criteria. Within a 150-km radius of the Mahantango Creek watershed, only eight HPD stations have record lengths of at least 50 years and a record completeness of 80% or more (Fig. S3). Of these eight stations, the highest level of completeness is 88%. Perhaps more importantly, all eight stations frequently experience years with greater than 40% missing data, with several stations having multiple instances where entire calendar years of precipitation data are missing (Fig. S3).


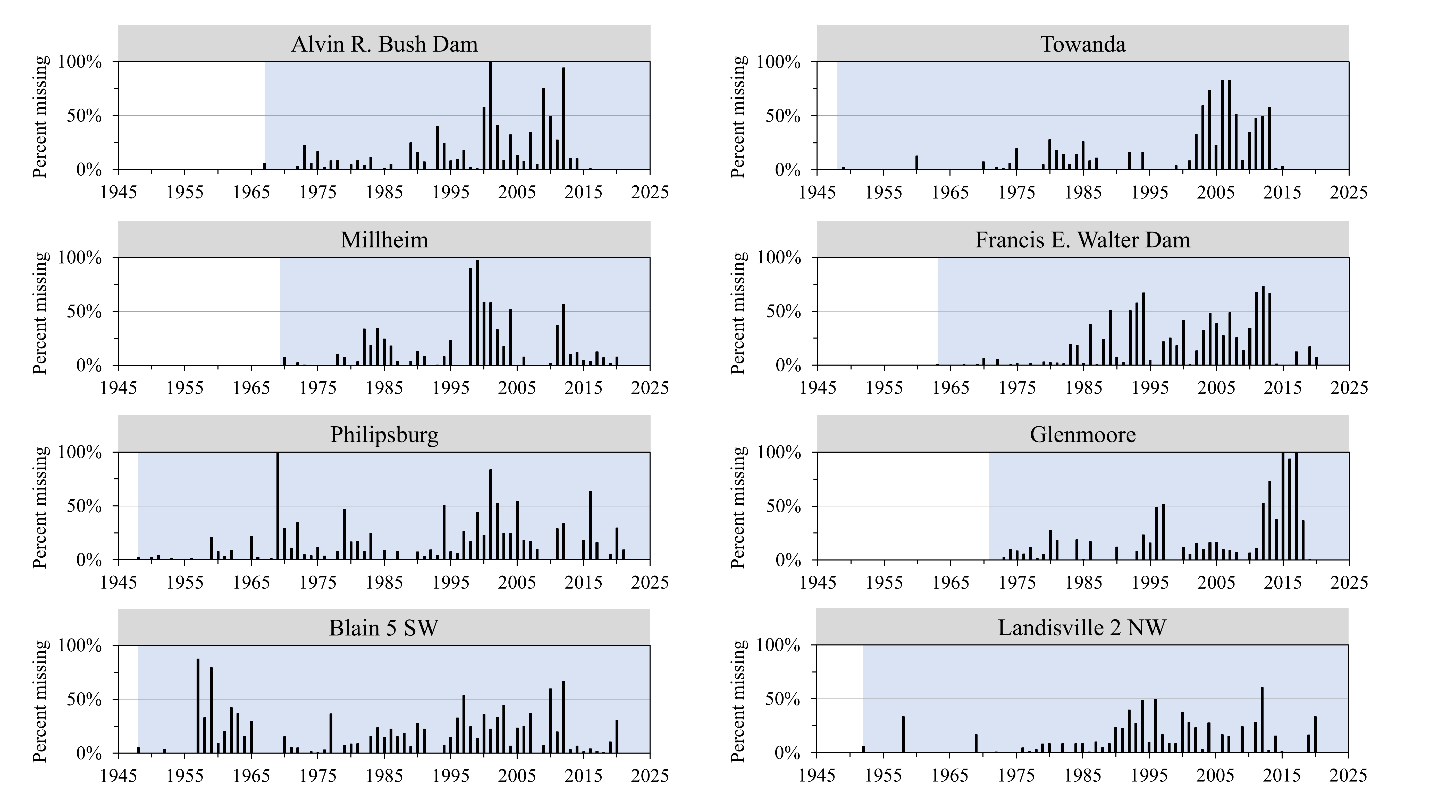


*Fig. S3. Graphs showing the percentage of missing data by year for the eight HPD rain gauges that lie within a 150-km radius of ARS’s long-term rain gauges in the Mahantango Creek watershed. The blue background in each graph indicates the period of record. Each of these gauges has at least 50 years of data and no more than 20% missing data over the entire period of record.*

In sum, the long-term record of 5-min precipitation from ARS’s WE-38 experimental watershed is highly unique, as there are few (if any) stations in the northeastern U.S. with 55 years of 5-min data that are at least 95% complete. Moreover, given the proximity of the three main gauges in the WE-38 watershed, there is the potential for creating a serially complete record from these data (see below). With the exception of USCRN, comparable national networks operated by NOAA’s NCEI – such as the COOP HPD network – typically report precipitation less frequently (currently every hour^13^) and also have data records with more than 5% of the records missing. Therefore, as Goodrich et al.^2^ note in the closing remarks of their paper on ARS watersheds, the 5-min rainfall records from WE-38 and similar such watersheds in ARS’s experimental watershed network have the potential to enable estimates of long-term rainfall trends at higher temporal resolutions than other networks of similar length.

S3. Infilling missing data and generating a serially complete record

There are many approaches to replacing missing values (a.k.a. gap filling), and these methods are reviewed by Paulhus and Kohler^26^, Eischeid et al.^27^, and Tang et al.^28^. The simplest approach – which we adopt in this paper – is the single best estimator (SBE) method. The SBE method requires a target gauge (e.g., Longman et al.^29^; Vicente-Serrano et al.^30^) and several nearby, similar gauges to infill missing values. In this study, RB-37 served as the target gauge, while RE-37 and MD-38 represented the primary and secondary neighboring gauges, respectively. We selected RB-37 as the target gauge because it had fewer and generally shorter data gaps than RE-37 (Table S1), the other long-term rain gauge with records dating back to 1968. Notably, RE-37 was inoperable for all of 1984, as well as during several three-month periods in 1983 and 1986.

To create a nearly serially-complete record, we used the SBE method to replace the missing data from the target station (RB-37) with the highest correlated predictor station ^27^. Because of the proximity of the gauges (maximum distance between gauges = 1.8 km), Pearson correlation coefficients between all gauge pairs exceeded 0.95 for daily rainfall totals, while correlations at hourly time scales were greater than 0.80, and correlations at 15-min intervals exceeded 0.55 (Table S3). Such high correlations further supported the use of nearest-neighbor methods to infill missing data gaps for the three measurement intervals (15 min, 1 h, and 1d) used in this study.

*Table S3. Pearson correlation coefficients between all gauge pairs in the WE-38 watershed. Correlation coefficients are shown for 5-min, 10-min, 15-min, 30-min, 1-h, and 1-d measurement intervals.*

| **Interval** | **ρ_RB-37, RE-37_** | **ρ_RB-37, MD-38_** | **ρ_RE-37, MD-38_** |
| --- | --- | --- | --- |
| 5 min | 0.30 | 0.32 | 0.34 |
| 10 min | 0.46 | 0.48 | 0.49 |
| 15 min | 0.56 | 0.58 | 0.60 |
| 30 min | 0.71 | 0.72 | 0.74 |
| 1 h | 0.82 | 0.83 | 0.84 |
| 1 d | 0.97 | 0.95 | 0.96 |

We note here that the RB-37 rain gauge had three periods of missing data that could not be filled using the primary (RE-37) or the secondary (MD-38) rain gauges. For instance, neither RB-37 nor RE-37 were operating during the latter part of Tropical Storm Agnes, which hit WE-38 on June 21–23, 1972; however, a nearby rain gauge (RE-40; in operation from 1966–1976), located 3.2 km east of WE-38 (see Fig. 1 in Engman et al.^31^), recorded the entire storm. As suggested by Buda et al.^9^, observations from RE-40 were used to infill the missing rainfall data from RB-37 and RE-37 (June 22, 1972 06:45 to the end of the storm on June 23, 1972 13:00). In addition, all three rain gauges (RB-37, RE-37, and MD-38) were down for a three-day period from August 1, 2004 18:35 to August 4, 2004 10:45. Observations from a short-term rain gauge (RD-36) in the southwest corner of the WE-38 watershed were used to infill missing 5-min rainfall data during this brief period of inoperability. Finally, the RB-37 and RE-37 rain gauges were inoperable from December 26, 1969 12:55 to January 13, 1970 14:00, and no other ARS rain gauges in Mahantango Creek had 5-min precipitation data for this time frame. Therefore, we were unable to impute the missing 5-min precipitation data for this 19-day period, which accounted for roughly 0.09% of the 55-year record. To shed some light on this issue, we extracted daily precipitation data from nClimGrid-Daily^32^ using the 5-km grid cell that included WE-38. Notably, nClimGrid-Daily reported one large rain event (precipitation = 31.8 mm) that occurred on December 26, 1969, and this event was largely captured by the RB-37 rain gauge before it malfunctioned at 12:55 that afternoon (recorded rainfall over the preceding 24-h = 22.9 mm). No other major rainfall events happened during the ensuing 18-day period ending on January 13, 1970, suggesting a low likelihood of missed rainfall extremes. Thus, we concluded that this 19-day period of missing data had minimal (if any) effects on the results we reported in the paper.

S4. Effects of record homogenization on 5-min precipitation data

To illustrate the effects of homogenizing the data based on Groisman et al.^33^, we estimated the simple 5-min intensity index for raw (unhomogenized) and homogenized time series of serially complete data. The simple 5-min intensity index represents the annual precipitation total divided by the annual number of 5-min intervals with precipitation (a.k.a. wet intervals, which are defined as any 5-min interval with precipitation > 0 mm). An increase in measurement precision, as with the rain gauges in WE-38, would increase the number of wet 5-min intervals and subsequently reduce the simple 5-min intensity index relative to the period that preceded the instrumentation change. As such, when we used the unhomogenized precipitation data to derive an annual time series of the simple 5-min intensity index, a Pettitt test^34^ revealed a significant change point in 1996/97 (Fig. S4). When we derived the simple 5-min intensity index from the homogenized data, the change point was no longer statistically significant (Fig. S4). This indicated that the serially complete 5-min precipitation data were appropriately homogenized to a consistent resolution of 2.54 mm for the entire period of record.


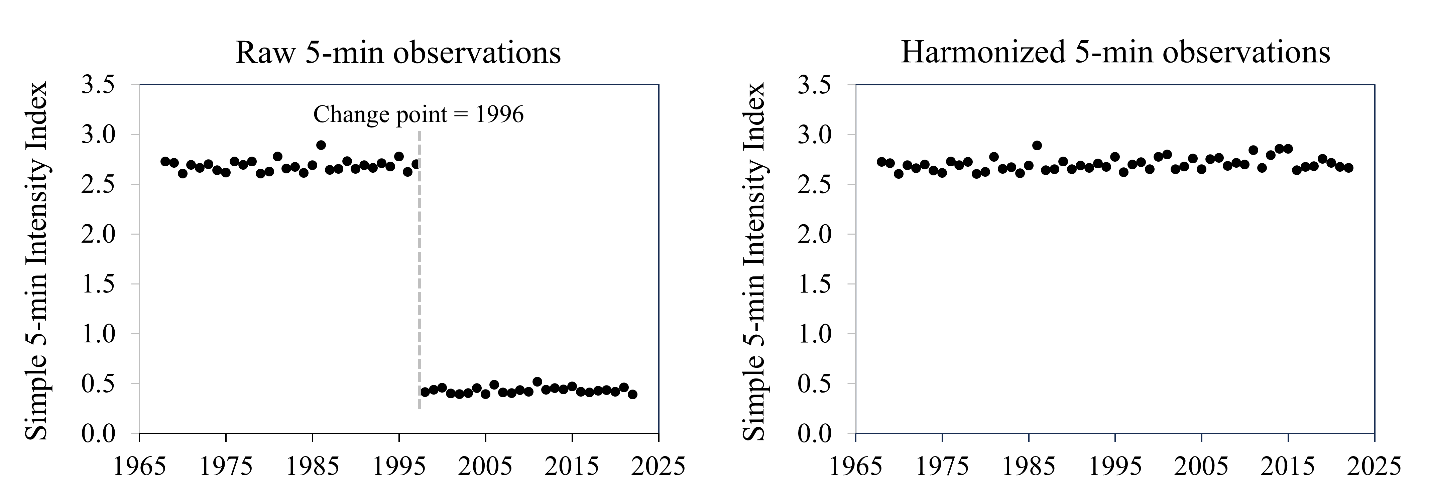


*Fig. S4. Graphs showing the simple 5-min intensity index for raw (unhomogenized) 5-min precipitation observations (left panel) and homogenized 5-min precipitation observations (right panel). Using the Pettitt test, a significant change point was detected in 1996 for the raw 5-min observations (p < 0.001), while no significant change point was detected for the homogenized data (p = 0.15).*

S5. Rx*15min*, Rx*1h*, and Rx*1d* trends via fixed intervals and moving windows

In our study, we used fixed intervals to aggregate 5-min precipitation data to 15-min, hourly, and daily accumulations in order to be consistent with other methods that relied on fixed interval data such as frequency analyses of extreme events. Even so, we recognized that fixed interval data often suffer from the “below the measurement interval truncation problem”, as detailed by Barbero et al.^24^. This issue can lead to a potential underestimation of annual and seasonal rainfall maxima, and possibly a misrepresentation of long-term trends in these time series. To address this issue, we used moving windows with lengths of 15 min, 1 h, and 24 h to estimate annual and seasonal Rx*15min*, Rx*1h*, and Rx*1d*. Comparisons of Rx*15min*, Rx*1h*, and Rx*1d* via fixed interval and moving window aggregations are shown in the box plots in Fig. S5. In line with Barbero et al.^35^, we found that moving window estimations of Rx*15min*, Rx*1h*, and Rx*1d* were roughly 1.2 times greater than those estimated by fixed intervals.


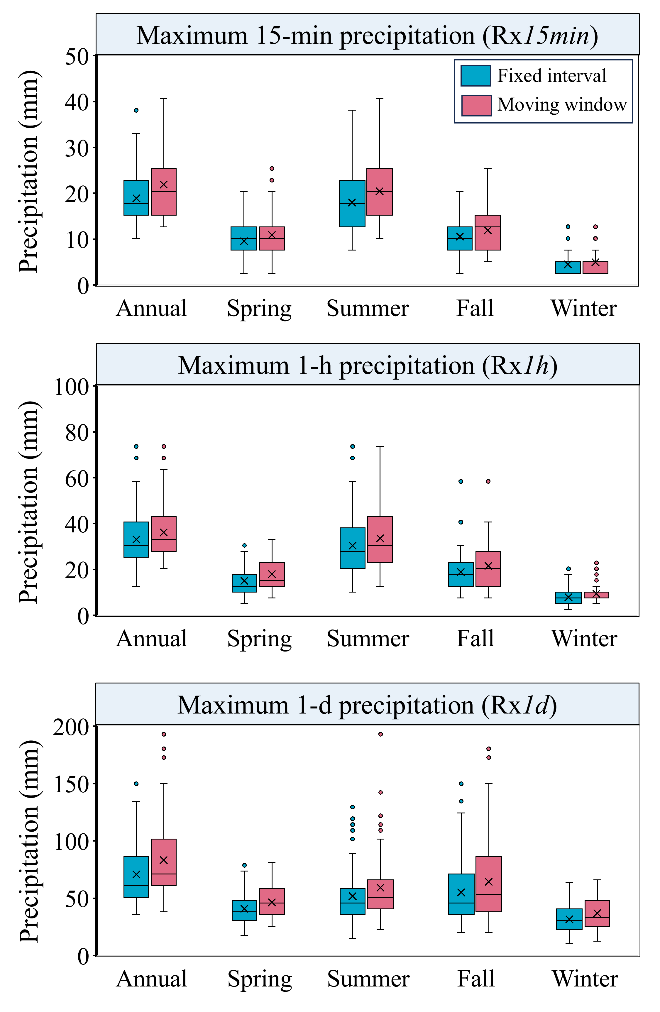


*Fig S5. Box plots showing the distribution of annual and seasonal maxima of 15-min (Rx15min; top panel), hourly (Rx1h; middle panel), and daily (Rx1d; lower panel) rainfall in the WE-38 watershed using fixed intervals (blue) and moving windows (red); long-term climatological averages are based on 55 years of data from 1968–2022. For each plot, the box indicates the interquartile range, the whiskers show the extreme values (1.5 times the interquartile range), the asterisks designate potential outliers (values greater or less than three times the interquartile range), the solid line depicts the median, and the “x” indicates the mean.*

We then sought to ascertain if moving window estimations of Rx*15min*, Rx*1h*, and Rx*1d* yielded different trends than the fixed interval data used in the paper. Briefly, we assessed trends in moving window summaries of Rx*15min*, Rx*1h*, and Rx*1d* using Mann-Kendall tests (Fig. S6) and nonstationary GEV models with a time-varying location parameter (Fig. S7).
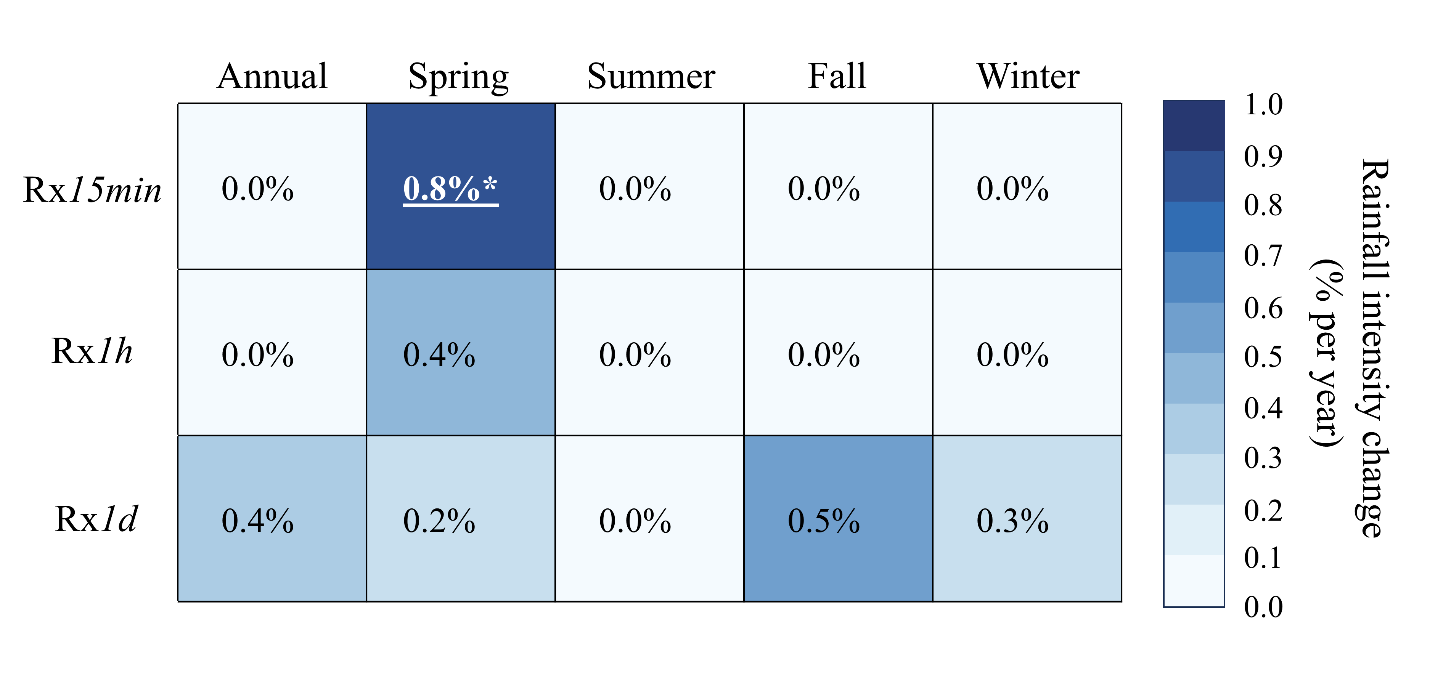


*Fig. S6. Annual and seasonal trends in the magnitude of Rx15min, Rx1h, and Rx1d based on Sen’s slope. Slopes are normalized over the mean value of each variable and expressed as a percent change per year. Statistically significant trends are determined with the Mann-Kendall test. Significant trends are underlined and bolded, with asterisks indicating the level of significance: * p ≤ 0.05; ** p ≤ 0.01; *** p ≤ 0.001.*

*
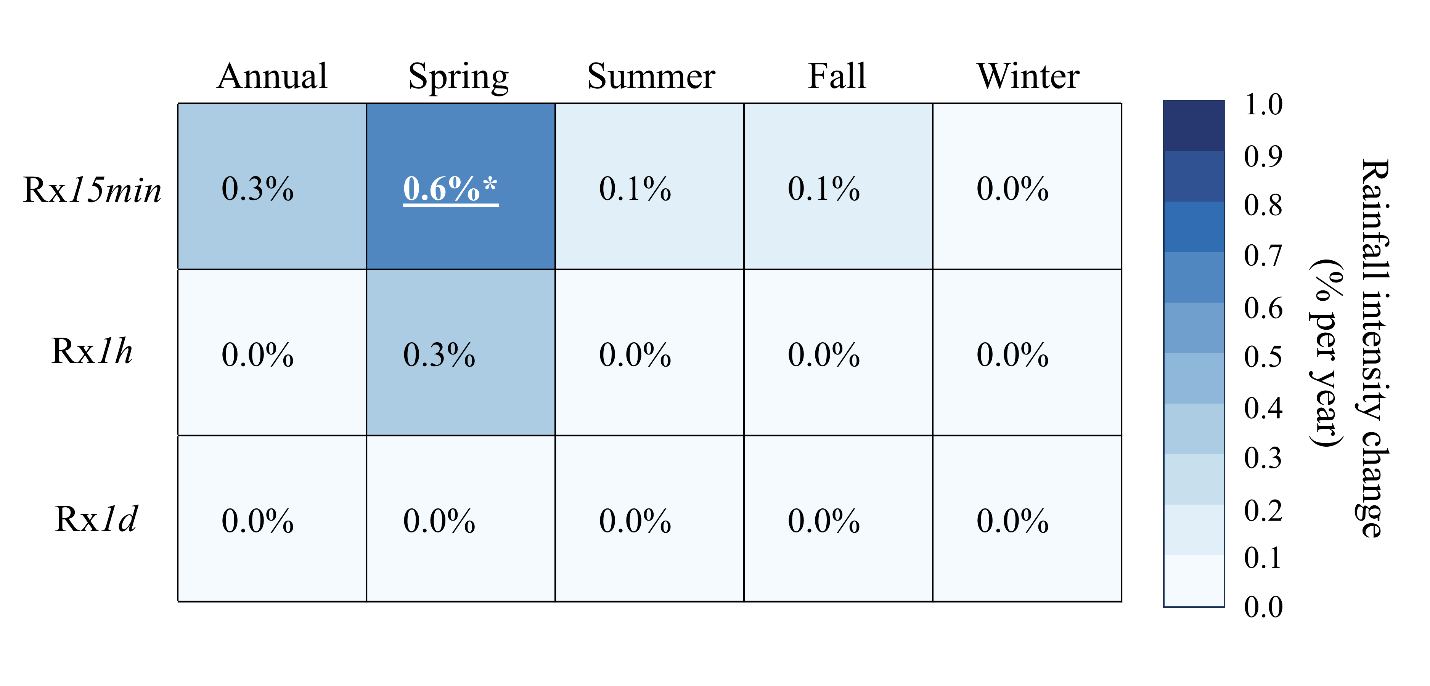
*

*Fig. S7. Annual and seasonal trends in the magnitude of Rx15min, Rx1h, and Rx1d using nonstationary GEV models with a time-varying location parameter. Slopes are normalized over the mean value of each variable and expressed as a percent change per year. Statistically significant trends are determined with the likelihood ratio test. Significant trends are underlined and bolded, with asterisks indicating the level of significance: * p ≤ 0.05; ** p ≤ 0.01; *** p ≤ 0.001.*

As we noted in the paper, the results of these trend tests generally agreed with the fixed interval results that were presented in Figs. 3 and 4. Most notably, Mann-Kendall tests using fixed interval data suggested that spring Rx*15min* increased at a rate of 0.9% per year, while the same tests applied to moving window aggregations indicated an increase of 0.8% per year. Using the nonstationary GEV models, both aggregation approaches agreed that Rx*15min* increased at a rate of 0.6% per year. Thus, even though moving window summaries of Rx*15min*, Rx*1h*, and Rx*1d* were larger than those from fixed interval data, the resultant trends were consistent.

S6. Air temperature and dew point temperature data and trends

In the WE-38 watershed, continuous 5-min measurements of air temperature (Ta, °C) and relative humidity (%) commenced in 1997, when a Campbell Scientific weather station was installed at the MD-38 gauging station^36^. Prior to 1997, Ta and relative humidity at the MD-38 station were usually recorded in the morning (between 0800 and 0900 h), with measurements of Ta restricted to daily maximums and daily minimums, and measurements of daily average relative humidity inferred from a hygrothermograph equipped with a continuous chart recorder. These earlier records contain numerous data gaps, especially the relative humidity measurements. Therefore, it was challenging to harmonize the earlier records with the more recent, continuous measurements from the climate station.

As indicated in the paper, our study sought to develop apparent scaling relationships between rainfall extremes and Td over the entire 55-yr study period from 1968–2022. However, given the lack of consistent relative humidity data prior to 1997, we were unable to reliably estimate Td. Therefore, we used daily mean values of Ta and Td from the ERA5-Land dataset^37^, as these data provided continuous, gap-free coverage back to 1968. To offer insight into the ability of ERA5-Land data to characterize local conditions in WE-38, we plotted observations of daily mean Ta and Td against values of daily mean Ta and Td obtained from ERA5-Land for the period from 1997–2022 (Fig. S8). The results of these analyses showed that the data from ERA5-Land provided a good representation of local climatic conditions in WE-38.


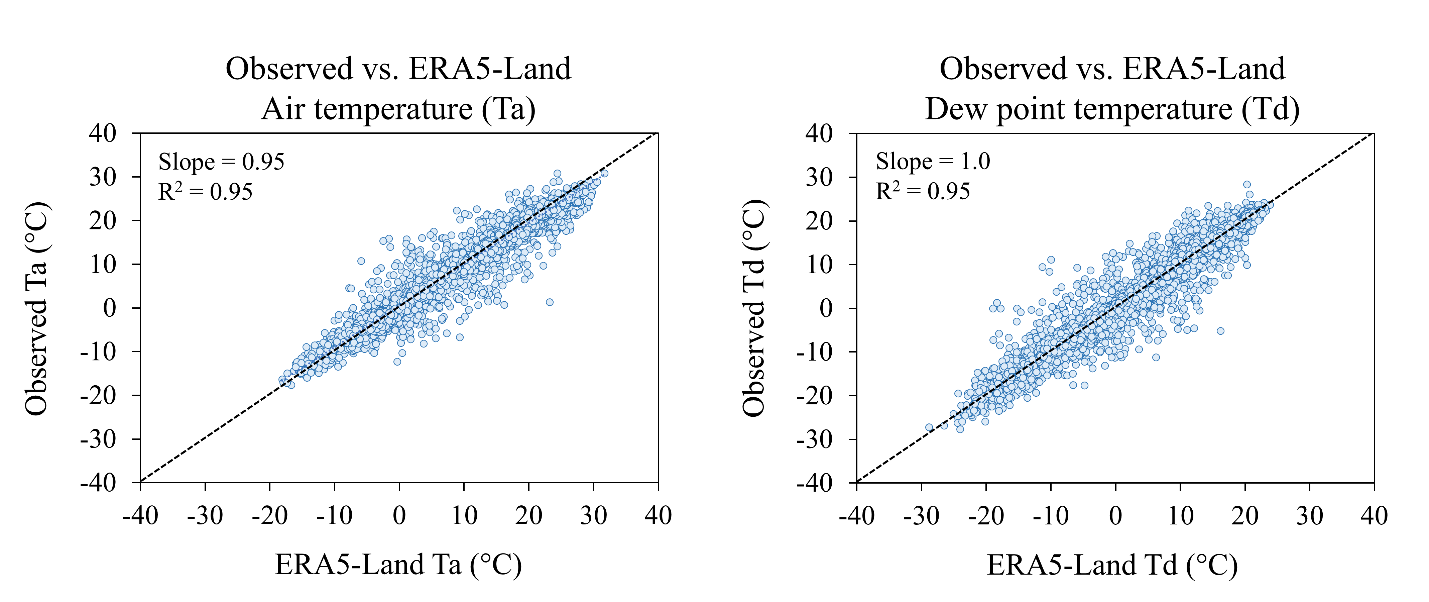


*Fig. S8. Plots of observed air temperature (Ta, °C) and dew point temperature (Td, °C) against Ta and Td from the ERA5-Land dataset for the period from 1997–2022. Dew point temperatures (Td) in the WE-38 watershed were estimated using the methods of Lawrence^38^.*

In support of the apparent scaling analyses of subhourly, hourly, and daily rainfall extremes, we also used the Mann-Kendall test and Sen’s slope estimator to evaluate trends in mean annual and mean seasonal values of Ta and Td (using ERA5-Land data) from 1968–2022 (Table S4).

*Table S4. Annual and seasonal trends (°C per year) in mean annual and mean seasonal Ta and Td based on Sen’s slope. Statistically significant trends are determined with the Mann-Kendall test. Significant trends are italicized, with asterisks indicating the level of significance: * p ≤ 0.05; ** p ≤ 0.01; *** p ≤ 0.001.*

| Variable | Annual | Spring | Summer | Fall | Winter |
| --- | --- | --- | --- | --- | --- |
| Ta (°C) | *0.03**** | *0.02** | *0.03**** | *0.02*** | *0.05**** |
| Td (°C) | *0.02*** | *0.02** | 0.01 | 0.01 | *0.04*** |

Results of these analyses showed that mean annual Ta increased by 0.03 °C per year, while increases in mean seasonal Ta ranged from 0.02 °C per year in spring and fall, to 0.03 °C per year in summer, to 0.05 °C per year in winter. These findings agreed results from Vose et al.^39^ showing that the fastest and most widespread warming was occurring in the winter.

In terms of Td, we found that mean annual Td increased by 0.02 °C per year. On a seasonal basis, the only seasons with statistically significant Td increases were spring and winter. Notably, the Td increase of 0.02 °C per year in the spring was generally consistent with earlier studies on Td trends in the U.S.^40,41^.

S7. Effects of ties on estimations of Rx*15min*P and Rx*1h*P

We used two metrics – Rx*15min*P and Rx*1h*P – to indicate the degree to which convection-driven storms were influencing trends in seasonal Rx*15min* and Rx*1h*. In the paper, we defined Rx*15min*P and Rx*1h*P as the fractional contributions of Rx*15min* and Rx*1h* to their respective daily rainfall totals^20,35,42^. It is important to note that while some years had only one value for Rx*15min* or Rx*1h*, other years had ties. Taking Rx*1h*P as an example, Pritchard et al.^20^ recommended using a randomly chosen value of Rx*1h* and its corresponding daily rainfall total to estimate Rx*1h*P for years with tied values of Rx*1h*. We adopted a different approach in this study, opting instead to use all tied values of Rx*1h* and their corresponding daily rainfall totals to estimate averaged values of Rx*1h*P for years with ties. We applied the same approach to estimating Rx*15min*P. To assess whether our approach yielded different results from the methods of Pritchard et al.^20^, we compared trends in Rx*15min*P and Rx*1h*P using the averaging method (averaging all tied values) and the random selection method (randomly selecting one value among all ties). As shown in Fig. S9 for the spring season, we found no appreciable difference in the magnitude or statistical significance of trends in Rx*15min*P and Rx*1h*P for either method. While we chose to highlight the spring because it was the season with the most evident trends in Rx*15min*P and Rx*1h*P, we found that the same general patterns held true for the other seasons (i.e., both methods were comparable). While these results suggested that either approach would be appropriate for estimating trends in Rx*15min*P and Rx*1h*P, we felt that including all the data (i.e., averaging ties rather than randomly selecting among tied data) was a more equitable approach to estimating these metrics.


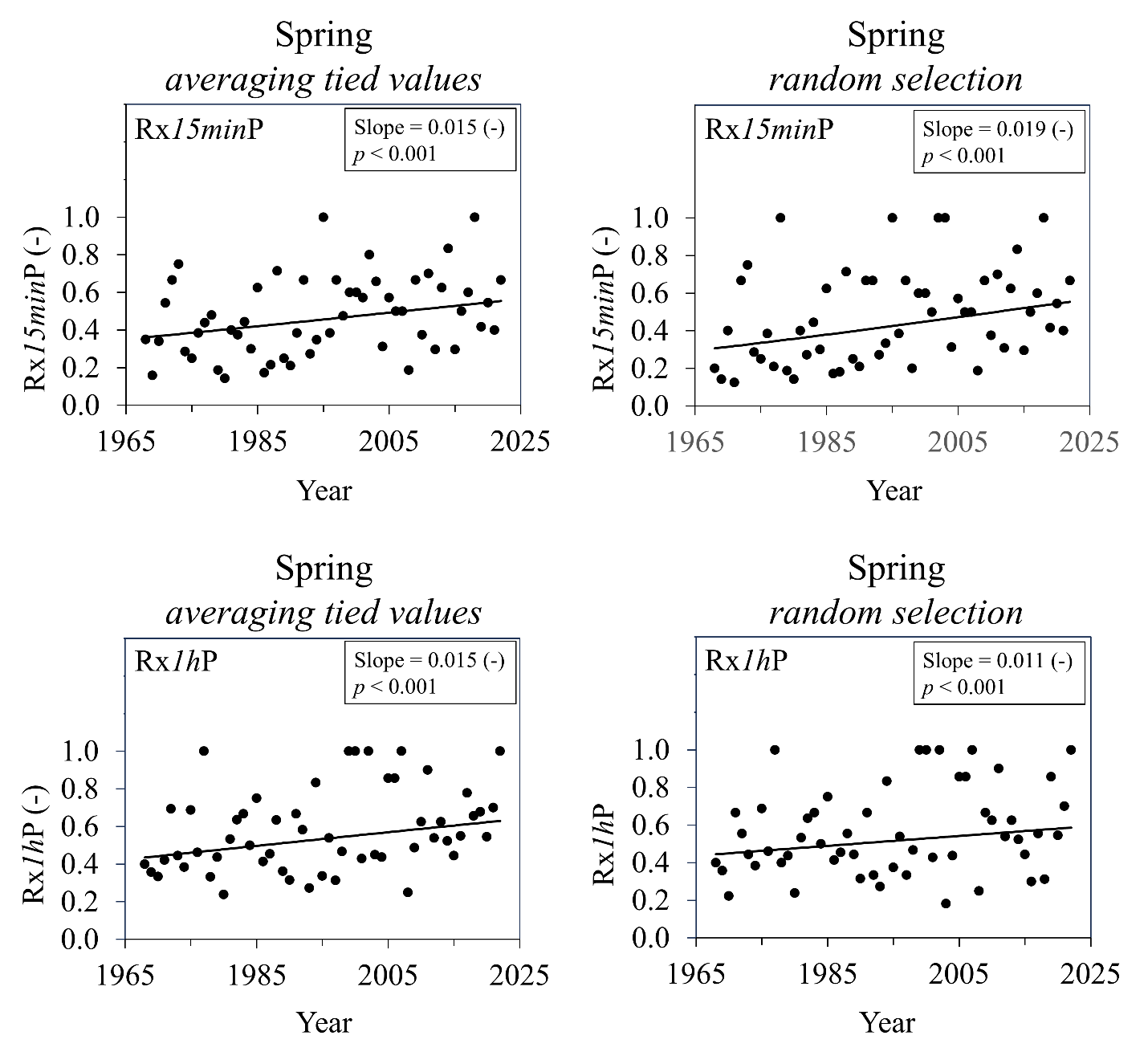


*Fig. S9. Trends in spring Rx15minP (top row) and spring Rx1hP (bottom row) showing effects of averaging tied values (left) and randomly selecting among tied values (right). Best fit regression lines are plotted using one-inflated beta regression^43^. Regression slopes are reported for statistically significant trends, along with the p-value. Note that the slopes of beta regression models indicate the change in the log odds of Rx15minP or Rx1hP being 1 given a unit increase in year.*

S7. References

1 Slaughter, C., W. & Richardson, C., W. Long-Term Watershed Research in USDA-Agricultural Research Service. *Water Resour. Impact* **2**, 28-32 (2000).

2 Goodrich, D. C. *et al.* The USDA-ARS Experimental Watershed Network: Evolution, Lessons Learned, Societal Benefits, and Moving Forward. *Water Resour. Res.* **57**, e2019WR026473 (2021). https://doi.org/https://doi.org/10.1029/2019WR026473

3 Harmel, R. D., King, K. W., Richardson, C. W. & Williams, J. W. Long-term precipitation analyses for the central Texas Blackland Prairie. *Trans. ASAE* **46**, 1381 (2003). https://doi.org/https://doi.org/10.13031/2013.15449

4 Bonta, J. V. Precipitation data considerations for evaluating subdaily changes in rainless periods due to climate change. *J. Soil Water Conserv.* **68**, 238-253 (2013). https://doi.org/10.2489/jswc.68.3.238

5 Goodrich, D. C. *et al.* Long-term precipitation database, Walnut Gulch Experimental Watershed, Arizona, United States. *Water Resour. Res.* **44** (2008). https://doi.org/https://doi.org/10.1029/2006WR005782

6 Starks, P. J. *et al.* Upper Washita River Experimental Watersheds: Meteorologic and Soil Climate Measurement Networks. *J. Environ. Qual.* **43**, 1239-1249 (2014). https://doi.org/https://doi.org/10.2134/jeq2013.08.0312

7 Hanson, C. L. Long-Term Precipitation Database, Reynolds Creek Experimental Watershed, Idaho, United States. *Water Resour. Res.* **37**, 2831-2834 (2001). https://doi.org/https://doi.org/10.1029/2001WR000415

8 Bosch, D. D., Sheridan, J. M. & Marshall, L. K. Precipitation, soil moisture, and climate database, Little River Experimental Watershed, Georgia, United States. *Water Resour. Res.* **43** (2007). https://doi.org/https://doi.org/10.1029/2006WR005834

9 Buda, A. R. *et al.* US Department of Agriculture Agricultural Research Service Mahantango Creek Watershed, Pennsylvania, United States: Long-term precipitation database. *Water Resour. Res.* **47** (2011). https://doi.org/10.1029/2010wr010058

10 Sadler, E. J., Sudduth, K. A., Drummond, S. T., Vories, E. D. & Guinan, P. E. Long-Term Agroecosystem Research in the Central Mississippi River Basin: Goodwater Creek Experimental Watershed Weather Data. *J. Environ. Qual.* **44**, 13-17 (2015). https://doi.org/https://doi.org/10.2134/jeq2013.12.0515

11 Carr, J. C. Rain gage network reports, in Klingerstown, Pennsylvania: Agricultural Research Service Precipitation Facilities and Related Studies, USDA-ARS 41-176, edited by D.M. Hershfield, chap. 11, 83-87. (1971).

12 Bryant, R. B. *et al.* US Department of Agriculture Agricultural Research Service Mahantango Creek Watershed, Pennsylvania, United States: Physiography and history. *Water Resour. Res.* **47** (2011). https://doi.org/10.1029/2010wr010056

13 Lawrimore, J. H. *et al.* Quality Control and Processing of Cooperative Observer Program Hourly Precipitation Data. *J. Hydrometeorol.* **21**, 1811-1825 (2020). https://doi.org/https://doi.org/10.1175/JHM-D-19-0300.1

14 Wuertz, D., Lawrimore, J. & Korzeniewski, B. Cooperative Observer Program (COOP) Hourly Precipitation Data (HPD), Version 2.0. In: Information, NOAA National Centers for Environmental Information (Ed.). DOI:10.25921/p7j8-2170 (2018).

15 Diamond, H. J. *et al.* U.S. Climate Reference Network after One Decade of Operations: Status and Assessment. *Bull. Am. Meteorol. Soc.* **94**, 485-498 (2013). https://doi.org/https://doi.org/10.1175/BAMS-D-12-00170.1

16 National Weather Service (NWS). Automated Surface Observation System (ASOS) user's guide. NOAA, 61 pp. (1998).

17 Dai, A., Karl, T. R., Sun, B. & Trenberth, K. E. Recent Trends in Cloudiness over the United States: A Tale of Monitoring Inadequacies. *Bull. Am. Meteorol. Soc.* **87**, 597-606 (2006). https://doi.org/https://doi.org/10.1175/BAMS-87-5-597

18 Doesken, N., McKee, T. & Davey, C. Climate Data Continuity - What Have We Learned From The ASOS Automated Surface Observing System. *13th Conference on Applied Climatology*. American Meteorological Society, Portland, OR (2002).

19 Lewis, E. *et al.* GSDR: A Global Sub-Daily Rainfall Dataset. *J. Clim.* **32**, 4715-4729 (2019). https://doi.org/https://doi.org/10.1175/JCLI-D-18-0143.1

20 Pritchard, D. *et al.* An Observation-Based Dataset of Global Sub-Daily Precipitation Indices (GSDR-I). *Sci. Data* **10**, 393 (2023). https://doi.org/10.1038/s41597-023-02238-4

21 Kunkel, K. E., 2013. Uncertainties in Observed Changes in Climate Extremes. In: AghaKouchak, A., Easterling, D., Hsu, K., Schubert, S., Sorooshian, S. (Eds.), Extremes in a Changing Climate: Detection, Analysis and Uncertainty. Springer Netherlands, Dordrecht, pp. 287-307. (2013). https://doi.org/10.1007/978-94-007-4479-0_10

22 Villarini, G., Mandapaka, P. V., Krajewski, W. F. & Moore, R. J. Rainfall and sampling uncertainties: A rain gauge perspective. *J. Geophys. Res. Atmos.* **113** (2008). https://doi.org/https://doi.org/10.1029/2007JD009214

23 Kundzewicz, Z. W. & Robson, A. J. Change detection in hydrological records—a review of the methodology / Revue méthodologique de la détection de changements dans les chroniques hydrologiques. *Hydrol. Sci. J.* **49**, 7-19 (2004). https://doi.org/10.1623/hysj.49.1.7.53993

24 Barbero, R., Fowler, H. J., Lenderink, G. & Blenkinsop, S. Is the intensification of precipitation extremes with global warming better detected at hourly than daily resolutions? *Geophys. Res. Lett.* **44**, 974-983 (2017). https://doi.org/10.1002/2016gl071917

25 DeGaetano, A. T. & Tran, H. Recent Changes in Average Recurrence Interval Precipitation Extremes in the Mid-Atlantic United States. *J. Appl. Meteorol. Clim.* **61**, 143-157 (2022). https://doi.org/https://doi.org/10.1175/JAMC-D-21-0129.1

26 Paulhus, J. L. H. & Kohler, M. A. Interpolation of missing precipitation records. *Mon. Weather Rev.* **80**, 129-133 (1952). https://doi.org/https://doi.org/10.1175/1520-0493(1952)080<0129:IOMPR>2.0.CO;2

27 Eischeid, J. K., Pasteris, P. A., Diaz, H. F., Plantico, M. S. & Lott, N. J. Creating a Serially Complete, National Daily Time Series of Temperature and Precipitation for the Western United States. *J. Appl. Meteorol.* **39**, 1580-1591 (2000). https://doi.org/https://doi.org/10.1175/1520-0450(2000)039<1580:CASCND>2.0.CO;2

28 Tang, G. *et al.* SCDNA: a serially complete precipitation and temperature dataset for North America from 1979 to 2018. *Earth Syst. Sci. Data* **12**, 2381-2409 (2020). https://doi.org/10.5194/essd-12-2381-2020

29 Longman, R. J., Newman, A. J., Giambelluca, T. W. & Lucas, M. Characterizing the uncertainty and assessing the value of gap-filled daily rainfall data in Hawaii. *J. Appl. Meteorol. Clim.*  **59**, 1261-1276 (2020).

30 Vicente-Serrano, S. M., Beguería, S., López-Moreno, J. I., García-Vera, M. A. & Stepanek, P. A complete daily precipitation database for northeast Spain: reconstruction, quality control, and homogeneity. *Int. J. Climatol.* **30**, 1146-1163 (2010). https://doi.org/https://doi.org/10.1002/joc.1850

31 Engman, E. T., Parmele, L. H. & Gburek, W. J. Hydrologic impact of tropical storm Agnes. *J. Hydrol.* **22**, 179-193 (1974). https://doi.org/https://doi.org/10.1016/0022-1694(74)90103-6

32 Durre, I., Arguez, A., Schreck, C. J., Squires, M. F. & Vose, R. S. Daily High-Resolution Temperature and Precipitation Fields for the Contiguous United States from 1951 to Present. *J. Atmos. Ocean. Technol.* **39**, 1837-1855 (2022). https://doi.org/https://doi.org/10.1175/JTECH-D-22-0024.1

33 Groisman, P. Y., Knight, R. W. & Karl, T. R. Changes in Intense Precipitation over the Central United States. *J. Hydrometeorol.* **13**, 47-66 (2012). https://doi.org/10.1175/jhm-d-11-039.1

34 Pettitt, A. N. A Non-Parametric Approach to the Change-Point Problem. *J. Roy. Stat. Soc. Ser. C. (Appl. Stat.)* **28**, 126-135 (1979). https://doi.org/https://doi.org/10.2307/2346729

35 Barbero, R. *et al.* A synthesis of hourly and daily precipitation extremes in different climatic regions. *Weather Clim. Extrem.* **26**, 100219 (2019). https://doi.org/https://doi.org/10.1016/j.wace.2019.100219

36 Lu, H. *et al.* Long-term trends in climate and hydrology in an agricultural, headwater watershed of central Pennsylvania, USA. *J. Hydrol. Reg. Stud.* **4**, 713-731 (2015). https://doi.org/https://doi.org/10.1016/j.ejrh.2015.10.004

37 Muñoz-Sabater, J. *et al.* ERA5-Land: a state-of-the-art global reanalysis dataset for land applications. *Earth Syst. Sci. Data* **13**, 4349-4383 (2021). https://doi.org/10.5194/essd-13-4349-2021

38 Lawrence, M. G. The Relationship between Relative Humidity and the Dewpoint Temperature in Moist Air: A Simple Conversion and Applications. *Bull. Am. Meteorol. Soc.* **86**, 225-234 (2005). https://doi.org/10.1175/bams-86-2-225

39 Vose, R. S., Easterling, D. R., Kunkel, K. E., LeGrande, A. N., Wehner, M. F. Temperature changes in the United States. In: Climate Science Special Report: Fourth National Climate Assessment, Volume I [Wuebbles, D. J., Fahey, D. W., Hibbard, K. A., Dokken, D. J., Stewart, B. C., Maycock, T. K. (eds.)]. U.S. Global Change Research Program, Washington, D.C, USA, 185-206. (2017). https://www.doi.org/10.7930/J0N29V45

40 Brown, P. J. & DeGaetano, A. T. Trends in U.S. Surface Humidity, 1930–2010. *J. Appl. Meteorol. Clim.* **52**, 147-163 (2013). https://doi.org/https://doi.org/10.1175/JAMC-D-12-035.1

41 Robinson, P. J. Temporal trends in United States dew point temperatures. *Int. J. Climatol.* **20**, 985-1002 (2000). https://doi.org/https://doi.org/10.1002/1097-0088(200007)20:9<985::AID-JOC513>3.0.CO;2-W

42 Blenkinsop, S., Lewis, E., Chan, S. C. & Fowler, H. J. Quality-control of an hourly rainfall dataset and climatology of extremes for the UK. *Int. J. Climatol.* **37**, 722-740 (2017). https://doi.org/https://doi.org/10.1002/joc.4735

43 Ospina, R. & Ferrari, S. L. P. A general class of zero-or-one inflated beta regression models. *Comput. Stat. Data Anal.* **56**, 1609-1623 (2012). https://doi.org/https://doi.org/10.1016/j.csda.2011.10.005
